# Identification of novel locus associated with coronary artery aneurysms and validation of loci for susceptibility to Kawasaki disease

**DOI:** 10.1038/s41431-021-00838-5

**Published:** 2021-03-26

**Authors:** Clive Hoggart, Chisato Shimizu, Rachel Galassini, Victoria J. Wright, Hannah Shailes, Evan Bellos, Jethro A. Herberg, Andrew J. Pollard, Daniel O’Connor, Shing Wan Choi, Eleanor G. Seaby, Stephanie Menikou, Martin Hibberd, Neneh Sallah, David Burgner, Paul Brogan, Harsita Patel, Jihoon Kim, Adriana H. Tremoulet, Eeva Salo, Diana van Stijn, Taco Kuijpers, Jane C. Burns, Michael Levin, The International Kawasaki Disease Genetics Consortium, UK Kawasaki Disease Genetics Consortium, EUCLIDS Consortium

**Affiliations:** 1 Section of Paediatric Infectious Disease, Department of Infectious Disease, Imperial College London, London, UK; 2 Department of Genetics and Genomic Sciences, Icahn School of Medicine at Mount Sinai, New York, NY USA; 3 Department of Pediatrics, University of California San Diego, La Jolla, CA USA; 4 Oxford Vaccine Group, Department of Paediatrics, NIHR Oxford Biomedical Research Centre, University of Oxford, Oxford, UK; 5 London School of Hygiene and Tropical Medicine, London, UK; 6 Murdoch Children’s Research Institute, Royal Children’s Hospital Melbourne, Parkville, VIC Australia; 7 Institute of Child Health, University College London, London, UK; 8 Department of Biomedical Informatics, University of California San Diego, La Jolla, CA USA; 9 Rady Children’s Hospital San Diego, San Diego, CA USA; 10 Department of Paediatrics and Adolescent Medicine, Tyks University Hospital, Turku, Finland; 11 Department of Pediatric Immunology, Rheumatology & Infectious Diseases, Emma Children’s Hospital, Amsterdam University Medical Centre, University of Amsterdam, Amsterdam, The Netherlands; 12 Sanquin Research and Landsteiner Laboratory, Department of Blood Cell Research, Academic Medical Centre, University of Amsterdam, Amsterdam, The Netherlands

**Keywords:** Genetics research, Vasculitis, Aneurysm

## Abstract

Kawasaki disease (KD) is a paediatric vasculitis associated with coronary artery aneurysms (CAA). Genetic variants influencing susceptibility to KD have been previously identified, but no risk alleles have been validated that influence CAA formation. We conducted a genome-wide association study (GWAS) for CAA in KD patients of European descent with 200 cases and 276 controls. A second GWAS for susceptibility pooled KD cases with healthy paediatric controls from vaccine trials in the UK (*n* = 1609). Logistic regression mixed models were used for both GWASs. The susceptibility GWAS was meta-analysed with 400 KD cases and 6101 controls from a previous European GWAS, these results were further meta-analysed with Japanese GWASs at two putative loci. The CAA GWAS identified an intergenic region of chromosome 20q13 with multiple SNVs showing genome-wide significance. The risk allele of the most associated SNV (rs6017006) was present in 13% of cases and 4% of controls; in East Asian 1000 Genomes data, the allele was absent or rare. Susceptibility GWAS with meta-analysis with previously published European data identified two previously associated loci (*ITPKC* and *FCGR2A*). Further meta-analysis with Japanese GWAS summary data from the *CASP3* and *FAM167A* genomic regions validated these loci in Europeans showing consistent effects of the top SNVs in both populations. We identified a novel locus for CAA in KD patients of European descent. The results suggest that different genes determine susceptibility to KD and development of CAA and future work should focus on the function of the intergenic region on chromosome 20q13.

## Introduction

Kawasaki disease (KD) is now the leading cause of acquired heart disease in children in developed countries [1]. Although introduction of effective treatment with intravenous immunoglobulin (IVIG) reduces the incidence of coronary artery aneurysm (CAA), aneurysm formation continues to occur in a significant proportion of children. Recent reports suggest that using recommended *Z* score criteria for CAA, 10–30% of KD cases develop CAA [2–4]. The ongoing high rates of CAA, despite availability of treatment, have a number of possible explanations, including delayed diagnosis. However, most of the patients who develop CAA, including those who are diagnosed in the first 10 days of the illness, have evidence of coronary artery (CA) dilatation or CAA at initial presentation [3]. Others who go on to develop CAA may have inadequate response to IVIG and other agents and represent a group of patients with “resistant KD” who have a severe inflammatory process often poorly responsive to anti-inflammatory agents [5].

Attempts to identify patients at risk of CAA among patients presenting with KD have been only partially successful. Patients who have recurrent fever after IVIG (IVIG resistance) are known to have an increased risk of CAA [5]. However, clinical scoring systems that predict resistance to IVIG in Japan have been poorly predictive in other populations [6, 7]. Attempts to identify unique biomarkers that predict development of CAA have been largely unsuccessful [8, 9].

Genetic factors are now well established as playing a role in susceptibility to KD in both European descent and Asian populations [10]. Genes with validated associations with susceptibility to KD in multiple ethnic groups include *FCGR2A* and *ITPKC* [11, 12]. A genetic contribution to CAA seems likely as before effective therapy with IVIG was introduced, only 25–30% of affected children developed CAA [13].

A number of small genome-wide association studies (GWASs) and candidate gene studies have suggested a genetic contribution to CAA formation in KD but have not been validated [14–20]. Three GWASs for CAA have been published, but each had fewer than 50 cases and therefore very limited power [14–16]. In a GWAS comparing subjects who develop CAA with those who do not, we identified a new locus on chromosome 20 (Chr. 20) with genome-wide significance.

We further constructed a new KD susceptibility GWAS by pooling the newly genotyped KD cases and comparing with healthy controls. This cohort was meta-analysed with a previously reported European KD GWAS [11] and with publicly available GWAS summary data from Japanese KD susceptibility GWASs [12, 21].

## Methods

### Study design

**Fig. 1:**
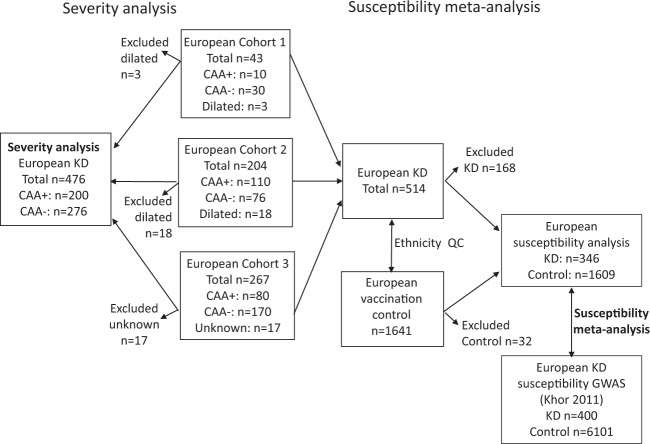
Study design. Flow diagram describes cohorts used in the study. Cohort 1: US subjects; Cohort 2: US and Finnish subjects; Cohort 3: UK and Dutch subjects. We first performed a severity analysis comparing KD subjects who developed CAA with those who did not. We next performed a new susceptibility GWAS comparing KD subjects and healthy controls and meta-analyzed this with GWAS data from the previously reported KD GWAS of European descent subjects [11]. Arrows leaving boxes denote subjects deleted from cohorts following more stringent ethnicity quality control or deleted because of missing information on CA status. Subjects who were classified as having dilated CA were excluded from the severity analysis but included for the susceptibility analysis. CA coronary artery, CAA coronary artery aneurysm.

The overall study design is shown in Fig. 1.

### Participant selection

Subjects meeting the AHA KD diagnostic criteria who presented to paediatric centres in USA, UK, Holland, and Finland between 2001 and 2018 were included. CAA were identified by echocardiography at each participating centre. Subjects were categorized by echocardiography using CA *Z* scores (internal diameter of the right CA or left anterior descending artery normalized for body surface area) as having giant CAA (*Z* score ≥ 10 or absolute dimension ≥8 mm). For the US and Dutch cohorts, lesser CAA were defined as *Z* ≥ 2.5. Patients scored as dilated coronary arteries (*Z* score 2–2.5) were excluded from the CAA comparison but included in the susceptibility GWAS. In the UK, *Z* scores were not routinely used to assign aneurysm size prior to 2017. Therefore, CAA were described according to the Japanese Ministry of Health criteria based on absolute luminal diameters (Research Committee of the Japanese Society of Pediatric, 2014). The majority of UK patients were retrospectively recruited to the study either through local hospitals or through the UK KD Support Group, a family-based group.

Controls for the susceptibility analysis were 1609 healthy infants undergoing routine vaccination in the UK [22]. For validation and meta-analysis of the susceptibility GWAS, we used the genotype data from 400 KD cases and 6101 European controls described in Khor et al. [11], and summary data from KD susceptibility GWASs of individuals of Japanese ancestry [12, 21] at two putative loci.

### Genotyping and statistical analysis

A detailed description of genotyping quality control (QC), imputation, statistical analysis, heritability estimation and Hi-C are in an Online Supplement Information. Briefly, after QC, multidimensional scaling (MDS) was applied (www.cog-genomics.org/plink/1.9/) to identify a genetically homogeneous group of European descent subjects for both severity and susceptibility GWAS. The genotype data were merged with the 1000 Genomes (1000G) [23] data and MDS was applied to determine the ancestry of the KD subjects. All data were imputed using the Haplotype Reference Consortium (HRC) reference panel (http://www.haplotype-reference-consortium.org) [24, 25]. To account for both ethnic diversity and cryptic relatedness, genome-wide association testing used logistic regression mixed models as implemented by the GMMAT software [26]. The top ten MDS components and two indicator variables for genotyping batch were included as fixed effects in both the CAA GWAS and new susceptibility GWAS. GWAS of the Khor KD susceptibility data used logistic regression in Plink2 (www.cog-genomics.org/plink/2.0/) with the top ten MDS components as covariates and was subsequently meta-analysed with the new GWAS. Loci were defined by ±250 kb of the top SNV in the region. Allelic heterogeneity was explored at each locus by conditional analyses in which analyses were rerun for all SNVs in loci, conditioning in the top SNV in the region.

The MAGMA software [27] was used for gene tests. Linkage disequilibrium (LD) score regression [28] and GCTA [29, 30] were used for heritability analyses. The functional impact of variants of interest were explored by querying eQTL associations in relevant tissues: specifically, whole blood, arteries and the heart using the GTEx database (https://www.gtexportal.org/home/) and the online FUMA tool (https://fuma.ctglab.nl/) [31] was used to query RegulomeDB and CADD scores.

Genome-wide summary statistics for the CAA GWAS and KD susceptibility meta-analysis are available to download from the EBI GWAS Catalog (https://www.ebi.ac.uk/gwas/studies/GCST90013538 and https://www.ebi.ac.uk/gwas/studies/GCST90013537).

## Results

### Characteristics of the study population

The cohorts of CAA+ and CAA− subjects after QC and removal of non-European subjects are described in Table 1 and Fig. 1.

**Table 1:** Description of KD cohorts with and without coronary artery aneurysms used in this analysis.

Cohort	Source	Platform	CAA+: *n* (% male)			CAA−: *n* (% male)	Total: *n* (% male)
			Small–medium CAA	Giant CAA	CAA+ subtotal		
1	US	Illumina 1.2 million SNV array	10 (70)	0	10 (70)	30 (60)	40 (63)
2	US	Illumina HumanCoreExome array customized	63 (63)	35 (60)	98 (62)	40 (78)	138 (67)
	Finland		8 (63)	4 (75)	12 (67)	36 (67)	48 (67)
3	UK	Illumina HumanCoreExome array	46 (70)	1 (100)	47 (70)	122 (65)	169 (66)
	Holland		21 (71)	12 (92)	33 (79)	48 (46)	81 (59)
Total			148 (67)	52 (69)	200 (68)	276 (63)	476 (65)

### QC of the genomic data

Supplementary Fig. 1A shows the MDS plot of the selected subjects by cohort and CAA status. Despite the relative genetic heterogeneity, there was an even spread by CAA status. The individuals clustered to the right of this figure were recruited in Finland. Supplementary Fig. 1B shows the MDS plot of KD subjects pooled with 1000G subjects and indicates those KD subjects determined to be of European ancestry who were used for the GWAS.

### Severity GWAS

**Fig. 2:**
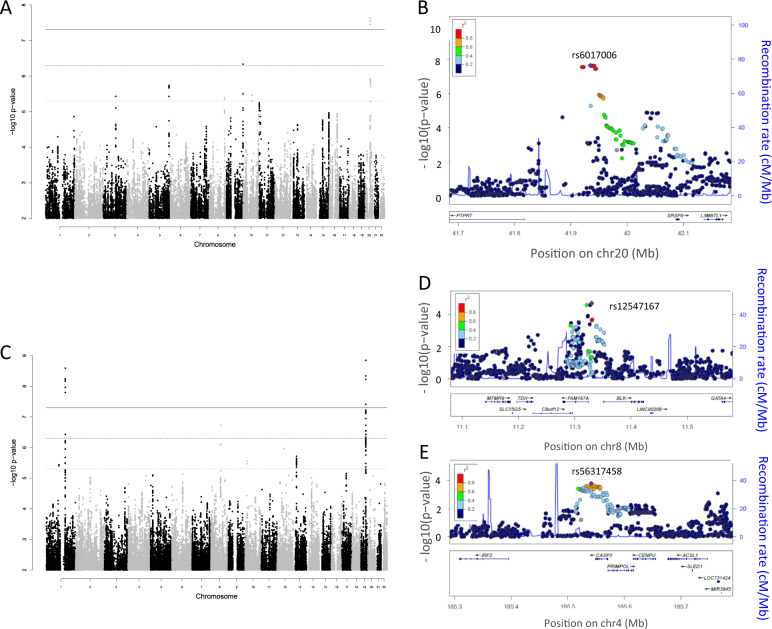
Severity and susceptibility GWAS results. **A** Manhattan plot for CAA GWAS (severity GWAS) showing genome-wide significant locus in intergenic region, Chr. 20 and five regions with association *p* < 5 × 10^−6^ (*UNC5B*, *MAN1B1* and *DOCK2* and two in intergenic regions). **B** Regional association plot of Chr. 20 intergenic SNV. **C** Manhattan plot for susceptibility GWAS showing confirmation of genome-wide significant SNVs in *IPTKC* and *FCGR2A*. **D** Regional association plots for previously published variants (*FAM167A-BLK*). **E** Regional plot of the CASP3 region associated with susceptibility. CAA coronary artery aneurysm.

After QC, 4,873,589 genotyped and imputed SNVs remained for the severity GWAS (CAA+ vs CAA−) and the resultant Manhattan plot is shown in Fig. 2A. The variance inflation factor (1.001) indicated that population stratification had been adequately accounted for (Supplementary Fig. 2). A single region on Chr. 20 spanning 41.9198–41.9449 Mb was identified for which 15 SNVs showed genome-wide significant differences between KD subjects with and without CAA (*p* < 5 × 10^−8^) (Table 2 and Supplementary Table 1). The top associated SNV rs6017006 had a *p* value of 2.3 × 10^−8^ and odds ratio for the minor risk allele A (vs G) of 5.0 (95% CI 2.8–9.0). The regional association plot for this association is shown in Fig. 2B. The imputation quality score *R*^2^ for rs6017006 was 0.996, 0.923 and 0.954 in Cohorts 1, 2 and 3 (see Fig. 1 and Supplementary Methods), respectively. The frequency of the A allele of rs6017006 was 13 and 4% in subjects with and without CAA, respectively (Table 2). Amongst East Asian populations sampled by the 1000G project, the risk allele A is only observed in the Dai population of western China. In the other global populations covered by 1000G, the risk allele frequency is 1% in Africans, 5% in European descent, 3% in Americans and 3% in South Asians. The OR for giant CAA (giant CAA vs CAA−) was 4.0 (95% CI 1.5–10.7) comparable to the whole CAA+ group (see above). To ensure this association was not driven by population stratification arising from the cluster of Finnish samples, the analysis was rerun with these samples removed (11 cases, 35 controls), this gave a *p* value for rs6017006 of 3.5 × 10^−9^, OR = 6.1 (95% CI 3.2–11.6).

**Table 2:** Top variants in loci associated with CAA formation with *p* < 5 × 10^−6^.

SNV	Chr	Position^a^	Gene region	Allele		Alt. allele frequencies			
				Ref	Alt	CAA (+)	CAA (−)	OR (95% CI)	*p*
rs6017006	20	41934620	Intergenic	G	A	0.13	0.04	5.0 (2.8–9.0)	2.3 × 10^−8^
rs7871579	9	139990530	MAN1B1 intron	T	C	0.26	0.15	2.8 (1.8–4.2)	4.6 × 10^−7^
rs2449565	5	169135662	DOCK2 intron	A	G	0.75	0.61	2.3 (1.6–3.3)	1.9 × 10^−6^
rs10762437	10	73058469	UNC5B intron	A	G	0.25	0.16	2.5 (1.7–3.8)	3.5 × 10^−6^
rs35932034	3	106262305	Intergenic	T	C	0.03	0.11	0.2 (0.09–0.4)	3.7 × 10^−6^
rs1989051	8	129294118	Intergenic	T	C	0.69	0.57	2.2 (1.6–3.1)	4.0 × 10^−6^

ORs were calculated using a Wald test, and *p* values were calculated using a Score test. All effects are relative to the alternate allele, ref/alt alleles are defined by the HRC reference panel.

*Chr.* chromosome, *CAA* coronary artery aneurysm, *OR* odds ratio.

^a^GRCh37/hg19 position.

In addition to this region on Chr. 20, we identified five other loci (top SNV) showing suggestive significance (*p* < 5 × 10^−6^). (Table 2 and Supplementary Fig. 3A–E). These included a Chr. 9 region containing *MANB1*, a Chr. 5 region containing *DOCK2*, a Chr. 10 region containing *UNC5B* and intergenic regions on Chr. 3 and 8. The regional association plots for the six loci indicated that allelic heterogeneity is unlikely, this was confirmed by conditional analyses, which resulted in no SNVs reaching suggestive significance.

Supplementary Table 2 shows the top 50 genes from the MAGMA test, the most significant gene was *CDH3* with *p* = 8.3 × 10^−6^. The only SNV to show significant eQTL association (*p* < 5 × 10^−5^) was rs7871579 in *MAN1B1*, which is significantly associated with the expression of *MAN1B1-AS1* in arteries (aorta, tibial), heart (coronary, left ventricle and atrial appendage) and whole blood. *UAP1L1, SAPCD2, DPP7* and *NPDC1* also had significant eQTL associations with rs7871579 (Supplementary Table 3). None of the other variants had an eQTL association at *p* < 5 × 10^−5^. Two intergenic SNVs on Chr. 20 and one intronic SNV on *DOCK2*, which were in LD with rs6017006 on Chr. 20 and rs2449565 on *DOCK2*, respectively, had high CADD deleterious scores. However, the RegulomeDB score and chromatin state for biological evidence to be a regulatory element were not strong for these SNVs (Supplementary Table 4).

### Exploring the function of the Chr. 20 lead SNV

To explore how an intergenic region might affect the expression of genes involved in CAA formation, we analysed published data using chromosomal conformational capture (3C) methodology on a genome-wide scale (Hi-C analysis) [32]. Conformational capture methods identify genomic regions that interact with each other in three-dimensional space and that might affect gene function or expression of genes that are not in linear proximity with the region of interest. Hi-C did not identify any interactions within 1 Mb of the identified SNV. However, there were interactions identified at greater distance (Supplementary Table 5). While long range interactions are less likely to be reproducible, *PLCB1*, the most significant interaction, was implicated as a risk locus for CAA in a Taiwanese study [33].

### Susceptibility GWAS

There were 5,784,964 genotyped and imputed SNVs passing QC in the new and Khor cohorts. The Manhattan plot for the KD susceptibility meta-analysis of these GWAS cohorts is shown in (Fig. 2C) and quantile–quantile plots for the individual GWASs and meta-analyses are shown in Supplementary Fig. 4A–C. The analysis identified two loci (top SNV ±250 kb) meeting genome-wide significance and two new loci with suggestive significance (*p* < 5 × 10^−6^). Table 3 shows results for the top SNV at each locus. Regional association plots for the loci in Table 3 are shown in Supplementary Fig. 5. Conditional analyses resulted in no SNVs reaching suggestive significance suggesting allelic heterogeneity was unlikely.

**Table 3:**
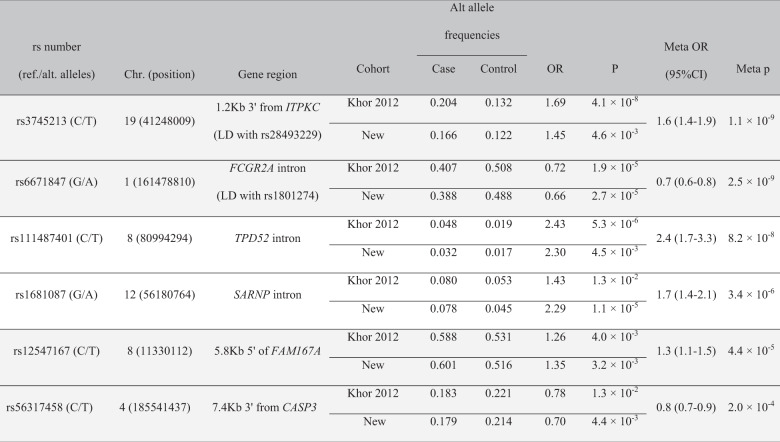
Variants associated with susceptibility to KD in order of descending significance.

All effects are relative to the alternate allele, ref/alt alleles are defined by the HRC reference panel. Shaded genes: previously reported in other ethnic groups. Highlighted variants validate previously published variants influencing susceptibility to KD in Asian and European descent cohorts.

*New* newly analysed European descent cohort in this paper, *Chr.* chromosome, position: GRCh37/hg19, *Meta* meta-analysis, *OR* odds ratio.

The most significant association in *ITPKC* (*p* = 1.1 × 10^−9^ for rs3745213) is in high LD with the previously reported variant, rs28493229, replicating findings from previous European and Asian genetic studies [11, 34, 35] (Supplementary Fig. 5A). The associated region in *FCGR2A* (*p* = 2.5 × 10^−9^ for rs6671847) is also in LD with rs1801274 (*R*^2^ = 0.89, D′ = 1) that has been associated with KD susceptibility in previous studies [11, 36] (Supplementary Fig. 5B).

The two additional SNVs showing suggestive evidence for association with KD susceptibility in our European meta-analysis were an intronic variant in TPD52 (*p* = 8.2 × 10^−8^, rs111487401) on Chr. 8 and an intronic variant in *SARNP* on Chr.12. (*p* = 3.4 × 10^−6^, rs1681087) (Supplementary Fig. 5C, D). Additional three SNVs showed suggestive evidence in the primary GWAS (score test followed by weighted *z* meta-analysis). However, these SNVs had *p* > 5 × 10^−6^ with Wald test and inverse-variance meta-analysis (Supplementary Table 6).

Supplementary Table 7 shows the top 50 genes associated with KD susceptibility from the MAGMA test using the meta-analysis results, the most significant gene was *CACNA2D3* with *p* = 2.4 × 10^−6^.

There were no significant eQTL associations for the SNVs in the two novel loci: rs111487401 in TPD52 on Chr. 8 and rs1681087 in *SARNP* on Chr.12 (Supplementary Table 3). However, these two SNVs were in LD with SNVs that had RegulomeDB score and chromatin state with biological evidence to be a regulatory element but their CADD scores were low (Supplementary Table 4).

### Analyses of previously associated loci

Querying of the NHGRI EBI GWAS Catalog for KD susceptibility associations identified eight SNVs (with non-ambiguous alleles) with a *p* value of <5 × 10^−8^ located in six loci (Supplementary Table 8). In addition to *ITPKC* and *FCGR2A*, two other loci showed nominal levels of significance in our European meta-analysis: rs2736340 in the *BLK-FAM167A* locus on Chr. 8 [37] (European meta *p* value = 3.3 × 10^−3^, GWAS of Chinese subjects *p* = 9 × 10^−10^) and rs2130392 in *CASP3-CENPU* locus on Chr. 4 [12] (European meta *p* value = 0.011, GWAS of Japanese subjects *p* = 3 × 10^−8^). The loci that did not replicate were *CD40* on Chr. 20 (rs1569723 [37]) and *HLA-DQB2, HLA-DOB* on Chr. 6 (rs2857151 [12]). Supplementary Table 9 shows our meta-analysis results for the other SNVs from the EBI GWAS Catalog with suggestive evidence for association with KD susceptibility.

Summary level data of *CASP3* and *BLK/FAM167A* susceptibility loci were available from two GWASs of Japanese subjects [12, 21] and were meta-analysed with our European meta-analysis results. Results from meta-analysing with Japanese summary data from the *CASP3* locus [12, 21] are shown in Supplementary Table 10. The most significantly associated SNV in the region was rs2720378 (European meta *p* = 2.5 × 10^−3^, Japanese *p* = 3.5 × 10^−9^, global meta *p* = 1.2 × 10^−10^, global meta OR = 0.741 (95% CI: 0.676, 0.812)). A test for heterogeneity of effects between studies showed no differences in effects between populations (*p* = 0.2). Regional association plots for the Japanese GWAS and the meta-analysis are shown in Supplementary Fig. 6A, B. The top SNV in the region in our study (rs56317458, Table 3 and Supplementary Fig. 5E) was not included in the Japanese study.

The results from meta-analysing with Japanese summary data from the *FAM167A-BLK* locus [12] are shown in Supplementary Table 11. The most significant SNV in the meta-analysis was rs35393613 (European meta *p* = 6.9 × 10^−4^, OR = 1.3, Japanese *p* = 3.6 × 10^−12^, OR = 1.8, global meta *p* = 5.2 × 10^−13^, global meta OR = 1.51 (95% CI: (1.35, 1.688)) [12]. There was evidence for a difference in effect sizes between populations (*p* = 0.02), this could be explained by differences in LD between rs35393613 and the causal variant in the European and Japanese populations. Regional association plots for the Japanese GWAS and the meta-analysis are shown in Supplementary Fig. 7A, B. The top SNV in our analysis was not included in the Japanese data (rs12547167, see Table 3 and Supplementary Fig. 5F).

Finally, in Supplementary Table 12, we report results from our study of SNVs reported in the first European GWAS for KD susceptibility [38]. None of the 37 overlapping SNPs were significant at *p* < 0.05.

### Cross-phenotype associations

Supplementary Table 13 shows comparison between studies of SNVs associated in the susceptibility and severity GWASs. Only rs10762437 (*UNC5B* intron) shows nominal cross-phenotype association. The top SNV rs12547167 at the *FAM167A* locus did not pass QC in the CAA GWAS, the SNV reported has *R*^2^ = 0.6 with it. Results are not shown for the *MAN1B* or *TPD52* as neither passed QC in the Khor GWAS.

### Heritability

Estimates of the heritability of CAA risk gave inconclusive results with very wide confidence intervals using both GCTA and LD score. This is likely because of the relatively small sample size of this GWAS. Assuming the prevalence of KD in individuals of European descent to be 2 in 10,000 [3], LD score estimated heritability on the liability scale in our population of European descent to be 0.119 (95% CI: 0.046, 0.191). The heritability in other ethnic groups could be quite different because of differences in genetic and/or environmental contributions to KD susceptibility. Assuming the odds ratios and minor allele frequencies shown in Table 3, the explained heritability on the liability scale for the top SNVs in *ITPKC*, *FCGR2A*, *FAM167A* and *CASP3* were 0.0026. 0.0033, 0.0019 and 0.0009, respectively, yielding a total of 0.008. These results suggest that there are many other causative variants contributing to risk of KD and that risk is likely the result of combined effect of multiple variants in each individual.

## Discussion

Our new GWAS pooling cases from UK, Holland, USA and Finland identified a region of Chr. 20 significantly associated with CAA. Previous GWAS and candidate gene studies have implicated a number of genes or gene regions in CAA development but these reports have been in cohorts with relatively small numbers of CAA cases and therefore limited power to identify significant associations [14–20].

We have identified a novel association between CAA formation and a SNV rs6017006 located in an intergenic region of chromosome 20 which is in LD with rs6030760 (19 kb 5′ upstream of rs6017006, *R*^2^ = 1) that alters the binding motif for CCCTC-binding factors (CTCF). CTCF and cohesin generate the unknotted loop of DNA where enhancer–promoter interactions occur during cellular differentiation [39]. To understand the functional significance of this variant in the context of the 3D genome, we performed Hi-C analysis using published data in human B‐lymphoblastoid cell lines as they provide the deepest coverage [32]. This analysis identified an association with *PLCB1*, which has been implicated in CAA formation in a Taiwanese GWAS [17]. However, as the association is 35 Mb distant from the identified SNV, there is less confidence in the robustness of this association [40–42]. Further studies are needed to evaluate the biological mechanisms by which the associated region influences development of CAA. It is noteworthy that the Chr. 20 variant does not influence susceptibility to KD, only CAA. Furthermore, comparison of SNVs associated with CAA and those associated with susceptibility showed no cross-phenotype associations, suggesting that children who develop CAA are a genetic subset of those who are susceptible.

Suggestive association was found between risk of CAA and five other loci (*p* < 5 × 10^−6^). One of these, *UNC5B*, is expressed on endothelial progenitor cells and is a receptor for netrin 4 with potential biological relevance to KD and CAA. Expression of *UNC5B* was shown to be essential for netrin 4-mediated neovascularization following ischaemic injury [43]. Another variant at rs7871579 is located in an intron of *MAN1B1* (mannosidase alpha class 1B member 1), 9 Kb 5′ of *MAN1B1-AS1* and 11.5 Kb 3′ of *UAP1L1*. This locus is reported as a quantitative trait locus (eQTL) with *MAN1B1-AS1* in multiple tissues/cells including blood, heart, CA and fibroblasts (*p* = 2.3 × 10^−6^–5.3 × 10^−21^) and with *UAP1L1* in blood (*p* = 5.1 × 10^−17^) (Supplementary Table 3). *MAN1B1-AS1* is a long non-coding gene that may regulate expression of *MAN1B1*, which is the enzyme that removes the terminal mannose residue from the middle branch of N-glycans [44]. *MAN1B1* also has a non-enzymatic function to prevent secretion of abnormally folded proteins [45]. Therefore, patients with *MAN1B1* deficiency have accumulation of hybrid-type glycans and some high mannose structures in serum IgG and other glycoproteins [46]. Previous data indicate that endogenous IgG molecules from KD patients have more fucose, galactose and sialylated N-glycans, compared to healthy children [47]. *UAP1L1* is also a glycosylation enzyme [48] and may influence T-cell development [49]. The variant on Chr. 5 (rs2449565) is located in an intron of *DOCK2* that influences activation, migration and proliferation of T cells, B cells, NK cells, dendritic cells and neutrophils [50]. A PheWAS using the GWAS Atlas (https://atlas.ctglab.nl) of the SNVs associated with CAA development and susceptibility (Tables 2 and 3) revealed no cross-phenotype associations at *p* < 5 × 10^−5^ except rs6671847 in *FCGR2A*, which was in LD with the previously reported exonic non-synonymous SNV rs1801274 (Supplementary Table 15).

Our new KD susceptibility GWAS and meta-analysis with published GWASs have confirmed associations with *ITPKC* and *FCGR2A* variants with KD susceptibility [11, 12]. Nominal association was also observed for previously published variants in regions near *FAM167A-BLK* (*p* = 4.4 × 10^−5^) and *CASP3* (*p* = 2.0 × 10^−4^). Meta-analysis with published data from Japan at these loci showed significance for the same SNVs across European and Japanese populations with consistent effects observed in both populations. The multi-ethnic meta-analyses of the *CASP3* and *FAM167A-BLK* loci of Asian and European populations narrowed the association peaks and hence have better localized the causal variants in these regions. For both loci, the top SNV in the analysis of the European data was not included in the Japanese data. However, the data support the hypothesis that the same causal variants at these loci are acting in both populations. In contrast, we could not replicate the genome-wide significant associations observed at the *CD40* and *HLA-DQB2-HLA-DOB* loci in East Asian populations in our European data.

Two new susceptibility loci with *p* < 5 × 10^−6^ were identified in our meta-analysis. The first, rs111487401, is in *TPD52*, which is highly expressed in mature B cells and binds to annexin V1 in a calcium-dependent manner [51]. This gene may be involved in the secretory functions of mature plasma cells. Polyclonal B cell activation is a prominent feature of KD and secretion of immunoglobulin molecules may be influenced by this protein. The second nominally associated variant, *SARNP*, is expressed in the heart and is involved in mRNA splicing and export. It is induced by thrombopoietin [52], which is highly expressed in acute KD [53]. These variants will require further replication.

The finding of an intergenic region on Chr. 20 that is significantly associated with development of CAA, but has no apparent influence on susceptibility, supports the hypothesis that different genes determine susceptibility to KD and development of CAA. There are likely other variants that contribute to CAA that can only be studied by comparing KD subjects with and without CAA. The functional significance of the intergenic locus on Chr. 20 should be a focus of future studies.

Limitations to our study include the lack of coverage of the X and Y chromosomes. The fact that males develop aneurysms more often than females suggests that risk alleles may reside on the sex chromosomes and should be investigated in future studies. This study focused only on risk alleles for CAA in a European descent cohort and future studies should address other ethnic groups. Although this is the largest GWAS focused on risk of CAA, the sample size was smaller than that in many GWAS. Given that only 14% of the subjects with CAA carry the risk allele on Chr. 20, it is clear that other loci that influence CAA remain to be discovered.

Finally, our findings may have relevance for the newly emerged childhood inflammatory multisystem syndrome temporally associated with exposure to SARS-CoV-2 (PIMS-TS, also called MIS-C) [54]. In countries experiencing large numbers of patients with COVID-19, there has been an increase in children meeting diagnostic criteria for KD, of whom 10–20% develop CAA [55, 56]. The genes and regions identified in our study as conferring risk of KD and CAA warrant exploration in the patients with PIMS-TS.

In summary, we identified a novel locus for CAA in KD patients using a robust dataset of European descent subjects. Comparing our pooled KD subjects with healthy controls confirmed several previously identified loci that contribute to KD susceptibility, but demonstrated that the CAA locus was not implicated in host susceptibility. These results suggest that different genes determine susceptibility to KD and development of CAA and future work should focus on the function of the intergenic region on chromosome 20q13.

## Supplementary information

Supplementary Information

Supplementary Table 2

Supplementary Table 4

Supplementary Table 7

Supplementary Table 12

Supplementary Table 15
