# Supplementary material for: Identification of novel locus associated with coronary artery aneurysms and validation of loci for susceptibility to Kawasaki disease
Source: Eur J Hum Genet. 2021 Mar 26;29(12):1734–44. doi: 10.1038/s41431-021-00838-5 (PMC7994355; doi:10.1038/s41431-021-00838-5)
Supplement: Supplementary file 1 — Supplementary Information [file 41431_2021_838_MOESM1_ESM.docx]

**Supplementary Information Appendix**

**Table of Contents**

Table of Contents ..................................................................................................................... 1

List of Investigators…………………………………...………………………………………3

Supplementary Methods ................................................................................................... 17-21

Supplementary Table 1 ............................................................................................................22

Supplementary Table 2 (Excel attached)................................................................................23

Supplementary Table 3 .......... ...........................................................................................24-25

Supplementary Table 4 (Excel attached)

Supplementary Table 5 ......................................................................................................27-28

Supplementary Table 6 ...........................................................................................................29

Supplementary Table 7 (Excel attached)

Supplementary Table 8 ......................................................................................................... 31

Supplementary Table 9 ......................................................................................................... 32

Supplementary Table 10 ....................................................................................................... 33

Supplementary Table 11 .................................................................................................. 34-36

Supplementary Table 12 (Excel attached)

Supplementary Table 13 ....................................................................................................... 38

Supplementary Table 14 ....................................................................................................... 39

Supplementary Table 15 (Excel attached)

Supplementary Figure 1 ....................................................................................................41-42

Supplementary Figure 2 ..........................................................................................................43

Supplementary Figure 3 ....................................................................................................44-46

Supplementary Figure 4 .........................................................................................................47

Supplementary Figure 5 ....................................................................................................48-50

Supplementary Figure 6 .........................................................................................................51

Supplementary Figure 7 .........................................................................................................51

Supplementary References ............................................................................................... 53-54

**List of investigators**

**International Kawasaki Disease Genetic Consortium:**

Rolando Cimaz, MD; Victoria Wright, PhD; David Burgner, MD; Taco W. Kuijpers, MD; Nagib Dahdah, MD; Chiea Chuen Khor, PhD; Sonia Davila, PhD; Martin Hibberd, PhD; Jane W. Newburger, MD; Anne Rowley, MD.; Michael Levin MD, Jane C Burns, MD, Chisato Shimizu MD

**Members of The UK Kawasaki disease genetic consortium**

Dept of Paediatrics, Imperial College London; Professor M Levin, Rachel Galassini , Dr Victoria Wright, Dr Jethro Herberg

Addenbrookes Hospital, Cambridge: Dr Y Singh (PI), J Bytham, J Sharp

Airedale General Hospital Dr P Bala (PI), A Kitching

Alder Hey children’s Hospital: Dr S Paulus (PI), Prof E Carol (PI), Dr B Larru (PI). S Wadeson, J Johnstone, R Jennings

Birmingham Children’s Hospital; Dr A Chickermane (PI), K Cotter

Bradford Royal Infirmary: Dr H Jepps (PI); T Booth, R Swingler

Bristol Royal Infirmary: Prof R Tulloh (PI); Karen Sheehan

Burton Hospital: Dr M Ahmed (PI), S Boswell, C Backhouse

Calderdale Royal Hospital Dr M Olabi (PI), KU Rahman (PI), S Kilroy, M Home

Durham & Darlington NHS Trust: Dr T Banerjee (PI), Dr G Nyamugunduru (PI), A Cowton, D Egginton

East Surrey Hospital; Dr M Jawad (PI)L Bailey

Evelina Children’s Hospital: Dr E Menson (PI)

Great Ormond Street Hospital, Dr P Brogan (PI), Y Glackin

Harrogate Hospital: Miss C Brunskill (PI)

Heartlands Hospital Dr S Hackett (PI), J Daglish

Hereford County Hospital Dr S Meyrick (PI), E Collins

Hull University Teaching Hospital, Mr D Bolton (PI)

Imperial College Healthcare NHS Trust; Dr J Herberg (PI), S Gormley, S Mustafa

Ipswich Hospital: Dr P Desai (PI) L Hunt

Kingston Hospital Dr T Chawatama (PI), Dr S Luck (PI), J Crooks, T O’Brien

Leeds General Infirmary Dr S O’Riordan (PI), N Balatoni, N Maher

Macclesfield General Hospital: Dr Chandrasekaran (PI), N Keenan

New Cross Hospital, Wolverhampton, Dr K Davies (PI), S Kempson, C Busby

North Manchester General Hospital: Dr E Odeka (PI), G O’Connor

North Tees & Hartlepool Dr I Haar (PI), G Osborne, H Walker

Northwick Park Hospital: Dr A Williams (PI)

Oldham Hospital Dr E Odeka (PI), L Woodward, C Rishton

Peterborough City Hospital; Dr V Puthi (PI), A Pearson, P Goodyear

Pinderfields General Hospital, Dr C Davidson (PI), Dr N De Vere (PI), G Castle

Royal Albert Edward Infirmary, Dr M Farrier (PI), N Pemberton

Royal Bolton Hospital: Dr S Misra (PI), C Fish, P Graham, J Henry

Royal Lancaster Infirmary, Dr A Olabi (PI) K Allison

Royal Shrewsbury Hospital: Dr A Kannivelu (PI), Mr J Jones (PI)

Royal Stoke University Hospital: Dr J Alexander (PI) E Roe, R Pringle, A Cope

Sheffield Children’s Hospital, Dr F Shackley (PI), S Gormley

South Tees Hospital: Dr R Kumar (PI), GMcilhinney, S Armstrong

St George’s Hospital, Tooting, Prof P Heath (PI), E Vitale, J Stuart

St Richard’s Hospital, Chichester: Dr N Brennan (PI), S Floyd

Stepping Hill Hospital, Stockport, Dr C Cooper, S Bennett

Tameside Hospital, Dr A Petkar (PI), Dr C Greenway (PI), W Hulse

The Royal Alexandra Hospital, Brighton; Dr K Fidler (PI), K Moscovici, S Sobowieck Kouman

The Royal Brompton Hospital, Dr F Franklin (PI), Dr M Bartsota

The Royal Cornwall Hospital, Dr N Venkata (PI), Dr A Prendiville (PI), Dr O Elmasry (PI), Mrs H Osborne (PI), G Craig, B Bromage

Torbay Hospital, Dr M Raman (PI), Ms P Fitzell (PI), H Bearne, J Palmer

UK Kawasaki Support Group S Davidson, N Clements

**EUCLIDS CONSORTIUM MEMBERS**

EUCLIDS consortium (www.euclids-project.eu) is composed by:

**Imperial College partner (UK)**

**Members of the EUCLIDS Consortium at Imperial College London (UK) Principal investigator and co-investigators**

Michael Levin (EUCLIDS Coordinator, CI)

Lachlan Coin, Stuart Gormley, Shea Hamilton, Jethro Herberg, Bernardo Hourmat, Clive Hoggart, Myrsini Kaforou, Vanessa Sancho-Shimizu, Victoria Wright

Consortium members at Imperial College

Amina Abdulla, Paul Agapow, Maeve Bartlett, Evangelos Bellos, Hariklia Eleftherohorinou, Rachel Galassini, David Inwald, Meg Mashbat, Stefanie Menikou, Sobia Mustafa, Simon Nadel, Rahmeen Rahman, Clare Thakker

**EUCLIDS UK Clinical Network**

Poole Hospital NHS Foundation Trust, Poole: Dr S Bokhandi (PI), Sue Power, Heather Barham

Cambridge University Hospitals NHS Trust, Cambridge: Dr N Pathan (PI), Jenna Ridout, Deborah White, Sarah Thurston

University Hospital Southampton, Southampton: Prof S Faust (PI), Dr S Patel (co-investigator), Jenni McCorkell.

Nottingham University Hospital NHS Trust: Dr P Davies (PI), Lindsey Crate, Helen Navarra, Stephanie Carter

University Hospitals of Leicester NHS Trust, Leicester: Dr R Ramaiah (PI), Rekha Patel

Portsmouth Hospitals NHS Trust, London: Dr Catherine Tuffrey (PI), Andrew Gribbin, Sharon McCready

Great Ormond Street Hospital, London: Dr Mark Peters (PI), Katie Hardy, Fran Standing, Lauren O’Neill, Eugenia Abelake

King’s College Hospital NHS Foundation Trust, London; Dr Akash Deep (PI), Eniola Nsirim

Oxford University Hospitals NHS Foundation Trust, Oxford Prof A Pollard (PI), Louise Willis, Zoe Young

Kettering General Hospital NHS Foundation Trust, Kettering: Dr C Royad (PI), Sonia White

Central Manchester NHS Trust, Manchester: Dr PM Fortune (PI), Phil Hudnott

**SERGAS Partner (Spain)**

Principal Investigators

Federico Martinón-Torres^1^

Antonio Salas^1,2^

GENVIP RESEARCH GROUP (in alphabetical order):

Fernando Álvez González^1^, Ruth Barral-Arca^1,2^, Miriam Cebey-López^1^, María José Curras-Tuala^1,2^, Natalia García^1^, Luisa García Vicente^1^, Alberto Gómez-Carballa^1,2^, Jose Gómez Rial^1^, Andrea Grela Beiroa^1^, Antonio Justicia Grande^1^, Pilar Leboráns Iglesias^1^ , Alba Elena Martínez Santos^1^, Federico Martinón -Torres^1^, Nazareth Martinón-Torres^1^, José María Martinón Sánchez^1^, Beatriz Morillo Gutiérrez^1^, Belén Mosquera Pérez^1^, Pablo Obando Pacheco^1^, Jacobo Pardo-Seco^1,2^, Sara Pischedda^1,2^, Irene Rivero-Calle^1^, Carmen Rodríguez-Tenreiro^1^, Lorenzo Redondo-Collazo^1^, Antonio Salas Ellacuriaga^1,2^, Sonia Serén Fernández^1^, María del Sol Porto Silva^1^, Ana Vega^1,3,^ Lucía Vilanova Trillo^1^.

^1^ Translational Pediatrics and Infectious Diseases, Pediatrics Department, Hospital Clínico Universitario de Santiago, Santiago de Compostela, Spain, and GENVIP Research Group (www.genvip.org), Instituto de Investigación Sanitaria de Santiago, Galicia, Spain.

1. Unidade de Xenética, Departamento de Anatomía Patolóxica e Ciencias Forenses, Instituto de Ciencias Forenses, Facultade de Medicina, Universidade de Santiago de Compostela, and GenPop Research Group, Instituto de Investigaciones Sanitarias (IDIS), Hospital Clínico Universitario de Santiago, Galicia, Spain
2. Fundación Pública Galega de Medicina Xenómica, Servizo Galego de Saúde (SERGAS), Instituto de Investigaciones Sanitarias (IDIS), and Grupo de Medicina Xenómica, Centro de Investigación Biomédica en Red de Enfermedades Raras (CIBERER), Universidade de Santiago de Compostela (USC), Santiago de Compostela, Spain

EUCLIDS SPANISH CLINICAL NETWORK:

Susana Beatriz Reyes^1^, María Cruz León León^1^, Álvaro Navarro Mingorance^1^, Xavier Gabaldó Barrios^1^, Eider Oñate Vergara^2^, Andrés Concha Torre^3^, Ana Vivanco^3^, Reyes Fernández^3^, Francisco Giménez Sánchez^4^, Miguel Sánchez Forte^4^, Pablo Rojo^5^, J.Ruiz Contreras^5^, Alba Palacios ^5^, Cristina Epalza Ibarrondo^5^, Elizabeth Fernández Cooke^5^, Marisa Navarro^6^, Cristina Álvarez Álvarez^6^, María José Lozano^6^, Eduardo Carreras^7^, Sonia Brió Sanagustín^7^, Olaf Neth^8^, Mª del Carmen Martínez Padilla^9^, Luis Manuel Prieto Tato^10^, Sara Guillén^10^, Laura Fernández Silveira^11^, David Moreno^12^.

1. Hospital Clínico Universitario Virgen de la Arrixaca; Murcia, Spain.
2. Hospital de Donostia; San Sebastián, Spain.
3. Hospital Universitario Central de Asturias; Asturias, Spain.
4. Complejo Hospitalario Torrecárdenas; Almería, Spain.
5. Hospital Universitario 12 de Octubre; Madrid, Spain.
6. Hospital General Universitario Gregorio Marañón; Madrid, Spain.
7. Hospital de la Santa Creu i Sant Pau; Barcelona, Spain.
8. Hospital Universitario Virgen del Rocío; Sevilla, Spain.
9. Complejo Hospitalario de Jaén; Jaén, Spain.
10. Hospital Universitario de Getafe; Madrid, Spain.
11. Hospital Universitario y Politécnico de La Fe; Valencia, Spain.
12. Hospital Regional Universitario Carlos Haya; Málaga, Spain.

**Members of the Pediatric Dutch Bacterial Infection Genetics (PeD-BIG) network (the Netherlands)**

*Steering committee:*

**Coordination:** R. de Groot ^1^, A.M. Tutu van Furth ^2^, M. van der Flier ^1^

**Coordination Intensive Care**: N.P. Boeddha ^3^, G.J.A. Driessen ^3^, M. Emonts ^3, 4, 5^, J.A. Hazelzet ^3^

**Other members**: T.W. Kuijpers ^7^, D. Pajkrt ^7^, E.A.M. Sanders ^6^ , D. van de Beek ^8^, A. van der Ende ^8^

**Trial coordinator**: H.L.A. Philipsen ^1^

**Local investigators (in alphabetical order)**

A.O.A. Adeel ^9^, M.A. Breukels ^10^, D.M.C. Brinkman ^11^, C.C.M.M. de Korte ^12^, E. de Vries ^13^ , W.J. de Waal ^15^, R. Dekkers ^15^, A. Dings-Lammertink ^16^ , R.A. Doedens ^17^, A.E. Donker ^18^, M. Dousma^19^, T.E. Faber ^20^, G.P.J.M. Gerrits^21^, J.A.M. Gerver ^22^, J. Heidema ^23^, J. Homan-van der Veen ^24^, M.A.M. Jacobs ^25^, N.J.G. Jansen ^6^, P. Kawczynski ^26^, K. Klucovska ^27^, M.C.J. Kneyber ^28^, Y. Koopman-Keemink ^29^, V.J. Langenhorst ^30^, J. Leusink ^31^, B.F. Loza ^32^, I.T. Merth ^33^, C.J. Miedema ^34^, C. Neeleman ^1^, J.G. Noordzij ^35^, C.C. Obihara ^36^ , A.L.T. van Overbeek – van Gils ^37^, G.H. Poortman ^38^,S.T. Potgieter ^39^, J. Potjewijd ^40^, P.P.R. Rosias ^41^, T. Sprong ^21^, G.W. ten Tussher ^42^, B.J. Thio ^43^, G.A. Tramper-Stranders ^44^, M. van Deuren ^1^, H. van der Meer ^2^, A.J.M. van Kuppevelt ^45^, A.M. van Wermeskerken ^46^, W.A. Verwijs ^47^, T.F.W. Wolfs ^4^.

1. Radboud University Medical Center – Amalia Children’s Hospital, Nijmegen, The Netherlands
2. Vrije Universiteit University Medical Center, Amsterdam, The Netherlands
3. Erasmus Medical Center – Sophia Children’s Hospital, Rotterdam, The Netherlands
4. Institute of Cellular Medicine, Newcastle University, Newcastle upon Tyne, United Kingdom
5. Paediatric Infectious Diseases and Immunology Department, Newcastle upon Tyne Hospitals Foundation Trust, Great North Children's Hospital, Newcastle upon Tyne, United Kingdom
6. University Medical Center Utrecht – Wilhelmina Children’s Hospital, Utrecht, The Netherlands
7. Academic Medical Center – Emma Children’s Hospital, University of Amsterdam, Amsterdam, The Netherlands
8. Academic Medical Center, University of Amsterdam, Amsterdam, The Netherlands
9. Kennemer Gasthuis, Haarlem, The Netherlands
10. Elkerliek Hospital, Helmond, The Netherlands
11. Alrijne Hospital, Leiderdorp, The Netherlands
12. Beatrix Hospital, Gorinchem, The Netherlands
13. Jeroen Bosch Hospital, ‘s-Hertogenbosch, The Netherlands
14. Diakonessenhuis, Utrecht, The Netherlands
15. Maasziekenhuis Pantein, Boxmeer, The Netherlands
16. Gelre Hospitals, Zutphen, The Netherlands
17. Martini Hospital, Groningen, The Netherlands
18. Maxima Medical Center, Veldhoven, The Netherlands
19. Gemini Hospital, Den Helder, The Netherlands
20. Medical Center Leeuwarden, Leeuwarden, The Netherlands
21. Canisius-Wilhelmina Hospital, Nijmegen, The Netherlands
22. Rode Kruis Hospital, Beverwijk, The Netherlands
23. St. Antonius Hospital, Nieuwegein, The Netherlands
24. Deventer Hospital, Deventer, The Netherlands
25. Slingeland Hospital, Doetinchem, The Netherlands
26. Refaja Hospital, Stadskanaal, The Netherlands
27. Bethesda Hospital, Hoogeveen, The Netherlands
28. University Medical Center Groningen, Beatrix Children’s hospital, Groningen, The Netherlands
29. Haga Hospital – Juliana Children’s Hospital, Den Haag, The Netherlands
30. Isala Hospital, Zwolle, The Netherlands
31. Bernhoven Hospital, Uden, The Netherlands
32. VieCuri Medical Center, Venlo, The Netherlands
33. Ziekenhuisgroep Twente, Almelo-Hengelo, The Netherlands
34. Catharina Hospital, Eindhoven, The Netherlands
35. Reinier de Graaf Gasthuis, Delft, The Netherlands
36. ETZ Elisabeth, Tilburg, The Netherlands
37. Scheper Hospital, Emmen, The Netherlands
38. St. Jansdal Hospital, Hardewijk, The Netherlands
39. Laurentius Hospital, Roermond, The Netherlands
40. Isala Diaconessenhuis, Meppel, The Netherlands
41. Zuyderland Medical Center, Sittard-Geleen, The Netherlands
42. Westfriesgasthuis, Hoorn, The Netherlands
43. Medisch Spectrum Twente, Enschede, The Netherlands
44. St. Franciscus Gasthuis, Rotterdam, The Netherlands
45. Streekziekenhuis Koningin Beatrix, Winterswijk, The Netherlands
46. Flevo Hospital, Almere, The Netherlands
47. Zuwe Hofpoort Hospital, Woerden, The Netherlands

***Swiss Pediatric Sepsis Study***

***Steering Committee****: Luregn J Schlapbach, MD, FCICM* *^1,2,3^, Philipp Agyeman, MD* *^1^,* *Christoph Aebi, MD ^1^, Christoph Berger, MD ^1^*

Luregn J Schlapbach, MD, FCICM ^1,2,3^, Philipp Agyeman, MD ^1^, Christoph Aebi, MD ^1^, Eric Giannoni, MD ^4,5^, Martin Stocker, MD ^6^, Klara M Posfay-Barbe, MD ^7^, Ulrich Heininger, MD ^8^, Sara Bernhard-Stirnemann, MD ^9^, Anita Niederer-Loher, MD ^10^, Christian Kahlert, MD ^10^, Paul Hasters, MD ^11^, Christa Relly, MD ^12^, Walter Baer, MD

^13^, Christoph Berger, MD ^12^ **for the Swiss Pediatric Sepsis Study**

1. Department of Pediatrics, Inselspital, Bern University Hospital, University of Bern, Switzerland
2. Paediatric Critical Care Research Group, Mater Research Institute, University of Queensland, Brisbane, Australia
3. Paediatric Intensive Care Unit, Lady Cilento Children’s Hospital, Children’s Health Queensland, Brisbane, Australia
4. Service of Neonatology, Lausanne University Hospital, Lausanne, Switzerland
5. Infectious Diseases Service, Lausanne University Hospital, Lausanne, Switzerland
6. Department of Pediatrics, Children’s Hospital Lucerne, Lucerne, Switzerland
7. Pediatric Infectious Diseases Unit, Children’s Hospital of Geneva, University Hospitals of Geneva, Geneva, Switzerland
8. Infectious Diseases and Vaccinology, University of Basel Children’s Hospital, Basel, Switzerland
9. Children’s Hospital Aarau, Aarau, Switzerland
10. Division of Infectious Diseases and Hospital Epidemiology, Children’s Hospital of Eastern Switzerland St. Gallen, St. Gallen, Switzerland
11. Department of Neonatology, University Hospital Zurich, Zurich, Switzerland
12. Division of Infectious Diseases and Hospital Epidemiology, and Children’s Research Center, University Children’s Hospital Zurich, Switzerland
13. Children’s Hospital Chur, Chur, Switzerland

***Liverpool Partner***

Principal Investigators

Enitan Carrol^1^

Stéphane Paulus ^1,2^

ALDER HEY SERIOUS PAEDIATRIC INFECTION RESEARCH GROUP (ASPIRE)

(in alphabetical order):

Hannah Frederick^3^, Rebecca Jennings^3^, Joanne Johnston^3^, Rhian Kenwright^3^

^1^ Department of Clinical Infection, Microbiology and Immunology, University of Liverpool Institute of Infection and Global Health, Liverpool, England

1. Alder Hey Children’s Hospital, Department of Infectious Diseases, Eaton Road, Liverpool, L12 2AP
2. Alder Hey Children’s Hospital, Clinical Research Business Unit, Eaton Road, Liverpool, L12 2AP

***Micropathology Ltd***

Colin G Fink^1,2^, Elli Pinnock^1^

^1^Micropathology Ltd Research and Diagnosis

^2^University of Warwick

***Newcastle partner***

Principle Investigator

Marieke Emonts^1,2^

Co-Investigator

Rachel Agbeko^1,3^

1. Institute of Cellular Medicine, Newcastle University, Newcastle upon Tyne, United Kingdom
2. Paediatric Infectious Diseases and Immunology Department, Newcastle upon Tyne Hospitals Foundation Trust, Great North Children's Hospital, Newcastle upon Tyne, United Kingdom
3. Paediatric Intensive Care Unit, Newcastle upon Tyne Hospitals Foundation Trust, Great North Children's Hospital, Newcastle upon Tyne, United Kingdom

***Gambia partner***

Suzanne Anderson: Principal Investigator and West African study oversight:

Fatou Secka: Clinical research fellow and study co-ordinator

Additional Gambia site team (consortium members):

Kalifa Bojang: co-PI

Isatou Sarr: Senior laboratory technician

Ngange Kebbeh: Junior laboratory technician

Gibbi Sey: lead research nurse Medical Research Council Clinic

Momodou Saidykhan: lead research nurse Edward Francis Small Teaching Hospital

Fatoumata Cole: Data manager

Gilleh Thomas: Data manager

Martin Antonio: Local collaborator

***Austrian partner***

**PI:** Werner Zenz^1^

**Co-Investigators/Steering committee:**

Daniela S. Klobassa^1^, Alexander Binder^1^, Nina A. Schweintzger^1^, Manfred Sagmeister^1^

^1^University Clinic of Paediatrics and Adolescent Medicine, Department of General Paediatrics, Medical University Graz, Austria

**Austrian network, participating centres in Austria, Germany, Italy, Serbia, Lithuania, patient recruitment (in alphabetical order):**

Hinrich Baumgart^1^, Markus Baumgartner^2^, Uta Behrends^3^, Ariane Biebl^4^, Robert Birnbacher^5^, Jan-Gerd Blanke^6^, Carsten Boelke^7^, Kai Breuling^3^, Jürgen Brunner^8^, Maria Buller^9^, Peter Dahlem^10^, Beate Dietrich^11^, Ernst Eber^12^, Johannes Elias^13^, Josef Emhofer^2^, Rosa Etschmaier^14^, Sebastian Farr^15^, Ylenia Girtler^16^, Irina Grigorow^17^, Konrad Heimann^18^, Ulrike Ihm^19^, Zdenek Jaros^20^, Hermann Kalhoff^21^, Wilhelm Kaulfersch^22^, Christoph Kemen^23^, Nina Klocker^24^, Bernhard Köster^25^, Benno Kohlmaier^26^, Eleni Komini^27^, Lydia Kramer^3^, Antje Neubert^28^, Daniel Ortner^29^, Lydia Pescollderungg^16^, Klaus Pfurtscheller^30^, Karl Reiter^31^, Goran Ristic^32^, Siegfried Rödl^30^, Andrea Sellner^26^, Astrid Sonnleitner^26^, Matthias Sperl^33^, Wolfgang Stelzl^34^, Holger Till^1^, Andreas Trobisch^26^ , Anne Vierzig^35^, Ulrich Vogel^12^, Christina Weingarten^36^, Stefanie Welke^37^, Andreas Wimmer^38^, Uwe Wintergerst^39^, Daniel Wüller^40^, Andrew Zaunschirm^41^, Ieva Ziuraite^42^, Veslava Žukovskaja^42^

^1^Department of Pediatric and Adolescence Surgery, Division of General Pediatric Surgery, Medical University Graz, Austria

^2^Department of Pediatrics, General Hospital of Steyr, Austria

^3^Department of Pediatrics/Department of Pediatric Surgery, Technische Universität München (TUM), Munich, Germany

^4^Department of Pediatrics, Kepler University Clinic, Medical Faculty of the Johannes Kepler University, Linz, Austria

^5^Department of Pediatrics and Adolesecent Medicine LKH Villach, Austria

^6^Department of Pediatrics and Adolescent Medicine and Neonatology, Hospital Ludmillenstift, Meppen, Germany

^7^Hospital for Children's and Youth Medicine, Oberschwabenklinik, Ravensburg, Germany

^8^Department of Pediatrics, Medical University Innsbruck, Austria

^9^Clinic for Paediatrics and Adolescents Medicine, Sana Hanse-Klinikum Wismar, Germany

^10^Departement of Pediatrics, Medical Center Coburg, Germany

^11^University Medicine Rostock, Department of Pediatrics (UKJ), Rostock, Germany

^12^Department of Pulmonology, Medical University Graz, Austria

^13^Institute for Hygiene and Microbiology, University of Würzburg, Germany

^14^Clinical Institute of Medical and Chemical Laboratory Diagnostics, Medical University Graz, Austria

^15^Department of Pediatric Orthopedics and Adult Foot and Ankle Surgery, Orthopedic Hospital Speising, Vienna, Austria

^16^Department of Paediatrics, Regional Hospital Bolzano, Italy

^17^Department of Pediatrics and Adolescent Medicine, General Hospital Hochsteiermark/Leoben, Austria

^18^Department of Neonatology and Paediatric Intensive Care, Children's University Hospital, RWTH Aachen, Germany

^19^Paediatric Intensive Care Unit, Department of Paediatric Surgery, Donauspital Vienna, Austria

^20^Department of Pediatrics, General Public Hospital, Zwettl, Austria

^21^Pediatric Clinic Dortmund, Germany

^22^Department of Pediatrics and Adolescent Medicine, Klinikum Klagenfurt am Wörthersee, Klagenfurt, Austria

^23^Catholic Children's Hospital Wilhelmstift, Department of Pediatrics, Hamburg, Germany

^24^Department of Pediatrics, Krankenhaus Dornbirn, Austria

^25^Children’s Hospital Luedenscheid, Maerkische Kliniken, Luedenscheid, Germany

^26^Department of General Paediatrics, Medical University Graz, Austria

^27^Department of Paediatrics, Schwarzwald-Baar-Hospital, Villingen-Schwenningen, Germany

^28^Department of Paediatrics and Adolescents Medicine, University Hospital Erlangen, Germany

^29^Department of Pediatrics and Adolescent Medicine, Medical University of Salzburg, Austria

^30^Paediatric Intensive Care Unit, Medical University Graz, Austria

^31^Dr. von Hauner Children's Hospital, Ludwig-Maximilians- Universitaet, Munich, Germany

^32^Mother and Child Health Care Institute of Serbia, Belgrade, Serbia

^33^Department of Pediatric and Adolescence Surgery, Division of Pediatric Orthopedics, Medical University Graz, Austria

^34^Department of Pediatrics, Academic Teaching Hospital, Landeskrankenhaus Feldkirch, Austria

^35^University Children’s Hospital, University of Cologne, Germany

^36^Department of Pediatrics and Adolescent Medicine Wilheminenspital, Vienna, Austria

^37^Department of Pediatric Surgery, Municipal Hospital Karlsruhe, Germany

^38^Hospital of the Sisters of Mercy Ried, Department of Pediatrics and Adolescent Medicine, Ried, Austria

^39^Hospital St. Josef, Braunau, Austria

^40^Christophorus Kliniken Coesfeld Clinic for Pediatrics, Coesfeld, Germany

^41^Department of Paediatrics, University Hospital Krems, Karl Landsteiner University of Health Sciences, Krems, Austria

^42^Children‘s Hospital, Affiliate of Vilnius University Hospital Santariskiu Klinikos, Lithuania

Genetic Determinants of Kawasaki Disease study (UK) study coordinator; Rachel Galassini

Addenbrookes Hospital, Cambridge: Dr Y Singh (PI), J Bytham

Airedale General Hospital PI Dr P Bala: A Kitching

Alder Hey children’s Hospital: Dr S Paulus (PI), Prof E Carol (PI), Dr B Larru (PI). S Wadeson, J Johnstone, R Jennings

Birmingham Children’s Hospital; Dr A Chickermane (PI), Cotter

Bradford Royal Infirmary: Dr H Jepps (PI); T Booth, R Swingler

Bristol Royal Infirmary: Prof R Tulloh (PI); Karen Sheehan

Burton Hospital: Dr M Ahmed (PI), S Boswell, C Backhouse

Calderdale Royal Hospital Dr M Olabi (PI), KU Rahman (PI), S Kilroy, M Home

Durham & Darlington NHS Trust: Dr T Banerjee (PI), Dr G Nyamugunduru (PI), A Cowton, D Egginton

East Surrey Hospital; Dr M Jawad (PI)L Bailey

Evelina Children’s Hospital: Dr E Menson (PI)

Great Ormond Street Hospital, Dr P Brogan (PI), Y Glackin

Harrogate Hospital: Miss C Brunskill (PI)

Heartlands Hospital Dr S Hackett (PI), J Daglish

Hereford County Hospital Dr S Meyrick (PI), E Collins

Hull University Teaching Hospital, Mr D Bolton (PI)

Imperial College Healthcare NHS Trust; Dr J Herberg (PI), S Gormley, S Mustafa

Ipswich Hospital: Dr P Desai (PI) L Hunt

Kingston Hospital Dr T Chawatama (PI), Dr S Luck (PI), J Crooks, T O’Brien

Leeds General Infimary Dr S O’Riordan (PI), N Balatoni, N Maher

Macclesfield General Hospital: Dr Chandrasekaran (PI), N Keenan

New Cross Hospital, Wolverhampton, Dr K Davies (PI), S Kempson, C Busby

North Manchester General Hospital: Dr E Odeka (PI), G O’Connor

North Tees & Hartlepool Dr I Haar (PI), G Osborne, H Walker

Northwick Park Hopsital: Dr A Williams (PI)

Oldham Hospital Dr E Odeka (PI), L Woodward, C Rishton

Peterborough City Hospital; Dr V Puthi (PI), A Pearson, P Goodyear

Pinderfields General Hospital, Dr C Davidson (PI), Dr N De Vere (PI), G Castle

Royal Albert Edward Infirmary, Dr M Farrier (PI), N Pemberton

Royal Bolton Hospital: PI Dr S Misra, C Fish, P Graham, J Henry

Royal Lancaster Infirmary, Dr A Olabi (PI) K Allison

Royal Shrewsbury Hospital: Dr A Kannivelu (PI), Mr J Jones (PI)

Royal Stoke University Hospital: Dr J Alexander (PI) E Roe, R Pringle, A Cope

Sheffield Children’s Hospital, Dr F Shackley (PI), S Gormley

South Tees Hospital: Dr R Kumar (PI), GMcilhinney, S Armstrong

St George’s Hospital, Tooting, Prof P Heath (PI), E Vitale, J Stuart

St Richard’s Hospital, Chichester: Dr N Brennan (PI), S Floyd

Stepping Hill Hospital, Stockport, Dr C Cooper, S Bennett

Tameside Hospital, Dr A Petkar (PI), Dr C Greenway (PI), W Hulse

The Royal Alexandra Hospital, Brighton; Dr K Fidler (PI), K Moscovici, S Sobowieck Kouman

The Royal Brompton Hospital, Dr F Franklin (PI), Dr M Bartsota

The Royal Cornwall Hospital, Dr N Venkata (PI), Dr A Prendiville (PI), Dr O Elmasry (PI), Mrs H Osborne (PI), G Craig, B Bromage

Torbay Hospital, Dr M Raman (PI), Ms P Fitzell (PI), H Bearne, J Palmer

UK Kawasaki Support Group S Davidson, N Clements

**Supplementary Methods**

**Genotyping quality control and imputation**

**Severity analysis**

Genotyping was performed using Illumina genotyping arrays. Samples collected through UCSD (USA) for Cohort 1 used the Illumina 1.2 million SNV array. Cohort 2 USA and Finland was typed on the Illumina Infinium bead chip platform at the Genome Institute of Singapore. The UK/Holland (Cohort 3) was genotyped on the Illumina Human Core Exome array at the Wellcome Trust Sanger Center.

Pre-imputation quality control (QC) and imputation were performed in each cohort separately. Pre-imputation sample QC removed duplicates, those with miss-matching genetic and recorded sex and samples with missing genotypes>5%. SNV QC removed SNVs with >5% missing genotypes and those that departed significantly from Hardy-Weinberg equilibrium with p<10^-6^. Additionally, in Cohort 2 for which parental genotype data was available for some samples, samples with biologically inconsistent parental genotype data were removed.

To identify an ethnically homogeneous group for analysis, genotype data was merged with 1000 Genomes (1000G) data and multidimensional scaling (MDS) was applied (as implemented in Plink 1.9). The majority of subjects mapped to European ancestry (**Supplementary Figure 1B in Supplement**). KD subjects who lay within the 1000G European cluster (as defined by the minima and maxima of MDS components 1 and 2 that captured all 1000G European subjects) were selected for analysis. The relationship matrix and MDS components were then recalculated for the selected KD subjects alone for use in subsequent analysis (**Supplementary Figure 1A in Supplement**). MDS and relationship matrix calculations used 108,654 genome-wide autosomal SNVs selected such that the maximum r^2^ between any two pairs of SNVs was less than 0.2, calculated in 200 SNV windows, shifted at 20 SNV intervals, as implemented in Plink 1.9.

Imputation used the Michigan Imputation server with the HRC reference panel, ShapeIT for phasing and minimac3 for imputation(ref. 1). Post-imputation QC removed SNVs with imputation quality score R^2^<0.8 or minor allele frequency < 5% in any of the three cohorts. The cohorts were then merged for analysis using Plink 2 and bcftools.

**Susceptibility analysis**

A case:control KD susceptibility GWAS data set was constructed by merging the genotype data from the KD cohorts described above passing QC with a cohort of genome-wide genotyped individuals recruited in the UK collected to assess response to vaccine as part of the EU funded EUCLIDS (European Union Life threatening Childhood Infection Study) study. Details of the vaccine cohort are reported in O'Connor et al.(ref. 2). Since cases and controls in this analysis were genotyped separately, stringent quality control was applied to each cohort before merging. Specifically, individuals with genotyping call rate<99% were removed and SNVs with missingness>1%, minor allele frequency<1% and those exhibiting significant departure from Hardy-Weinberg equilibrium (p<10^-5^) were removed.

All four genotype data sets were then merged across the common SNVs passing QC and MDS components were calculated for the merged data set. To ensure good ethnic matching of the cases and controls samples lying in clusters defined by MDS 1 v MDS 2 and MDS 3 v MDS 4 were selected. **Supplementary Figures 1C and D** in Supplement show plots of MDS 1 v MDS 2 and MDS 3 v MDS 4 colored by cohort and samples passing QC.

We had access to the genotype data used in the Khor KD susceptibility GWAS(ref. 3). To ensure the independence of the new susceptibility GWAS from the Khor data, and thus robust meta-analysis, samples in the new dataset with duplicates and related samples (to the level of first cousins) in the Khor data were removed. This was achieved by merging the samples passing QC with the Khor data at all overlapping SNVs and calculating the relationship matrix using plink1.9. Individuals in the new data set with inbreeding coefficient > 0.125 with an individual in the Khor data were removed. It was noted during the merging of all cohorts that there were pairs of related individuals in the Khor data, including pairs of half sibs. One from each of these pairs was removed using the rel-cutoff option in Plink with the default parameter of 0.05. This resulted in removing 5 cases and 151 controls from the Khor data. The top 10 MDS components were recalculated for the remaining samples in the new data set for inclusion in the GWAS.

To maximise the number of SNVs taken forward to imputation, three separate case control KD data sets were prepared for imputation, one for each KD data set with controls randomly assigned to each such that the case:control ratio was same across all three imputation cohorts (**Supplementary Table 14**). Imputation was run on the three cohorts using Eagle to phase the data and minimac4 for imputation with the HRC r1.1.2016 reference panel on the Michigan imputation server. Prior to imputation, SNVs with missingness >1%, minor allele frequency<1%, and those exhibiting significant departure from Hardy-Weinburg equilibrium (p<10^-5^) were removed. Plink2 and bcftools were used to process the data post-imputation.

The Khor data was imputed in an identical fashion.

**Statistical Analysis**

**GWAS for severity analysis**

After quality control, the selected European KD samples of 276 CAA- controls and 200 CAA+ cases represented a relatively ethnically diverse range of Europeans (**Figure 1**). To account for the ethnic diversity genome-wide association testing used logistic regression mixed models with the top ten MDS components included as fixed effects as implemented by the GMMAT software. Two binary covariates indicating the cohort of each sample were also included to control for possible batch effects. To maximise the power of the analysis we followed Yang et al (ref. 4) and used a relationship matrix in the mixed model calculated by removing the chromosome under test. All SNVs with minor allele frequency>5% and imputation quality score R^2^>0.8 were analyzed. Allelic dosages derived from the imputation probabilities of the three genotypes were used to account for the uncertainty of imputed genotypes.

**GWAS and meta-analysis for susceptibility**

For the susceptibility analysis, we compared the genotype data from the KD cases with genotype data from 1609 European healthy infants undergoing vaccination as reported(ref. 2). There was evidence of distant relationships within the cohort, to account for this we again used logistic regression mixed models as implemented in GMMAT, including covariates for the top 10 MDS components and two indicator variables for imputation run.

Logistic regression as implemented by Plink2 was used for the GWAS of the Khor data, analyzing SNVs with minor allele frequency>2% and imputation quality score R^2^>0.8. Initially the top four MDS dimensions were included as covariates in the logistic regression model mirroring the analysis strategy of Khor(ref. 3). This analysis resulted in a variance inflation factor of ƛ=1.04. Rerunning the GWAS including the top ten MDS components in the model resulted in ƛ=0.99. This analysis was the one taken forward to meta-analysis.

The two cohorts were meta-analyzed using weighted z-statistics(ref. 5), the inverse-variance method could not be used because GMMAT uses score tests for genome-wide association testing of SNV phenotype association because of its computational efficiency and thus does return odds ratios and standard errors. For all analyses, genotype dosages were used to account for uncertainty of imputed genotypes. GMMAT was rerun using a Wald test for SNVs of interest, these were SNVs with association p-values <5x10^-6^ and those reported in the literature, to enable inverse-variance meta-analysis and ORs to be reported. Wald and Score tests, although asymptotically equivalent, will always give slightly different results, however, it has been shown that for approximately equal numbers of cases and controls the Score test is more powerful(ref. 5), we therefore report the p-values from this test for the top SNVs in the CAA GWAS.

Previously reported associations with KD susceptibility were downloaded from the EBI GWAS Catalog (/www.ebi.ac.uk/gwas/) and were further inverse-variance meta-analyzed with our European meta-analysis results. Summary level data from the *CASP3* and *FAM167A-BLK* regions for a Japanese population were taken from Table 1 of Onouchi 2010(ref. 6) and Supplementary Table 3 of Onouchi 2012(ref. 7) respectively, this data was similarly meta-analyzed by the inverse-variance method.

**Heritability Estimation**

The LD Score software regression(ref. 8) was used to estimate heritability of susceptibility to KD using partitioned heritability (ref. 9). LD Score regression simply requires summary level GWAS data: SNV p-values, effect allele and effect direction. The meta-analysis results were used for the heritability of susceptibility to KD. As is typically for binary traits, we report heritability on the liability scale, which is invariant to prevalence. To calculate heritability on the liability scale from the raw LD Score output requires an estimate for the prevalence, we assumed this to be 2/10,000 for susceptibility to KD (ref. 10).

Heritability attributable to single SNVs on the liability scale was calculated using the formula in Lee et al (ref. 11) for R2 in ascertained case-control studies, the inputs for this calculation were the estimated effect sizes and allele frequencies from the meta-analysis.

**Hi-C methods**

Since our lead finding on chromosome 20 lies in an intergenic region, we interrogated potential distal interactions that might elucidate its mechanism of action. To that end, we used an established high-resolution genome-wide map of chromatin interactions based on the Hi-C protocol (ref. 12). Hi-C relies on proximity ligation followed by high-throughput sequencing, which effectively generates paired reads that align to distal genomic loci, corresponding to long-range DNA-DNA contacts. We focused on the densest available Hi-C map, which was generated from a human B-lymphoblastoid cell line and achieved kilobase resolution. From the publicly available dataset, we extracted the raw contact frequencies of the locus surrounding our SNV of interest (rs75202180). To account for potential biases in the raw frequencies (such as chromatin accessibility and alignment) we performed coverage normalisation, as per the original Hi-C analysis protocol. This process down weights short-distance interactions as they tend to be driven by linear proximity rather than true three-dimensional interaction. We then partitioned the rest of the genome into 10kb non-overlapping windows in search of loci that co-localize in 3D space with our region of interest.

**Supplementary Table 1.** All variants associated with CAA formation with p<5×10^-8^ at the Chromosome 20 locus. All effects are relative to the alternate allele, ref/alt alleles defined by the HRC reference panel.

|  |  |  | Allele | | Alt allele frequency | |  |  |
| --- | --- | --- | --- | --- | --- | --- | --- | --- |
| SNP | Chr | Position (hg19) | Reference | Alt. | CAA- | CAA+ | OR (95% CI) | p |
| rs8121349 | 20 | 41919800 | T | C | 0.05 | 0.13 | 5.132 (2.799,9.412) | 2.82E-08 |
| rs186774337 | 20 | 41921789 | A | G | 0.05 | 0.13 | 5.103 (2.79,9.335) | 2.76E-08 |
| rs6030760 | 20 | 41922755 | A | G | 0.05 | 0.13 | 5.062 (2.774,9.24) | 2.87E-08 |
| rs6017006 | 20 | 41934620 | G | A | 0.04 | 0.13 | 4.97 (2.752,8.978) | 2.30E-08 |
| rs140228249 | 20 | 41935339 | G | A | 0.04 | 0.13 | 4.966 (2.75,8.969) | 2.31E-08 |
| rs76267456 | 20 | 41936254 | T | A | 0.04 | 0.13 | 4.959 (2.747,8.952) | 2.32E-08 |
| rs80170284 | 20 | 41937903 | C | T | 0.04 | 0.13 | 4.954 (2.745,8.938) | 2.32E-08 |
| rs74740596 | 20 | 41938239 | C | T | 0.04 | 0.13 | 4.949 (2.744,8.927) | 2.32E-08 |
| rs111789291 | 20 | 41939963 | C | A | 0.04 | 0.13 | 4.943 (2.742,8.911) | 2.32E-08 |
| rs118008703 | 20 | 41940710 | G | A | 0.04 | 0.13 | 4.94 (2.741,8.905) | 2.32E-08 |
| rs13433114 | 20 | 41941449 | A | T | 0.04 | 0.13 | 4.936 (2.739,8.895) | 2.32E-08 |
| rs75202180 | 20 | 41941664 | A | G | 0.04 | 0.13 | 4.932 (2.738,8.886) | 2.32E-08 |
| rs74356518 | 20 | 41943242 | C | A | 0.05 | 0.13 | 4.73 (2.654,8.429) | 3.50E-08 |
| rs76212684 | 20 | 41944540 | C | A | 0.05 | 0.14 | 4.726 (2.653,8.421) | 3.53E-08 |
| rs77821729 | 20 | 41944898 | A | G | 0.05 | 0.13 | 4.727 (2.653,8.424) | 3.52E-08 |

**Supplementary Table 2.** Top 50 genes from a MAGMA gene test for association with CAA formation. Columns specify the gene name, genomic position, the number of SNVs included in the gene test, effective number of SNVs, test statistic and p-value.

Excel file attached

**Supplementary Table 3.** Association of the top CAA variants and KD susceptibility variants with gene expression in artery, heart, and whole blood with p<5x10^-5^in GTEx.

| Category | SNP ID | Region of SNP | SNP eQTL to | P-Value | NES (normalized effect size) | Tissue* |
| --- | --- | --- | --- | --- | --- | --- |
| CAA | rs6017006 | Intergenic | none |  |  |  |
|  | rs7871579 | MAN1B1 intron | UAP1L1 | 5.1E-17 | 0.44 | Whole Blood |
|  |  |  | MAN1B1-AS1 | 4.8E-16 | 0.43 | Artery - Aorta |
|  |  |  | SAPCD2 | 3.0E-13 | 0.39 | Whole Blood |
|  |  |  | DPP7 | 2.8E-12 | -0.25 | Artery - Tibial |
|  |  |  | NPDC1 | 2.1E-11 | -0.27 | Artery - Aorta |
|  |  |  | MAN1B1-AS1 | 2.8E-10 | 0.2 | Artery - Tibial |
|  |  |  | MAN1B1-AS1 | 2.9E-09 | 0.38 | Artery - Coronary |
|  |  |  | NPDC1 | 1.1E-07 | -0.16 | Artery - Tibial |
|  |  |  | MAN1B1-AS1 | 2.6E-07 | 0.25 | Heart - Left Ventricle |
|  |  |  | MAN1B1-AS1 | 7.9E-07 | 0.22 | Heart - Atrial Appendage |
|  |  |  | MAN1B1-AS1 | 2.3E-06 | 0.19 | Whole Blood |
|  | rs2449565 | DOCK2 intron | none |  |  |  |
|  | rs10762437 | UNC5B intron | none |  |  |  |
|  | rs35932034 | Intergenic | none |  |  |  |
|  | rs1989051 | Intergenic | none |  |  |  |

**Supplementary Table 3.: Continued.**

| Category | SNP ID | Region of SNP | SNP eQTL to | P-Value | NES (normalized effect size) | Tissue* |
| --- | --- | --- | --- | --- | --- | --- |
| /Susceptibility | rs3745213 | ITPKC | ITPKC | 4.20E-08 | -0.21 | Artery - Tibial |
|  |  |  | ITPKC | 1.80E-07 | -0.22 | Heart - Atrial Appendage |
|  |  |  | EGLN2 | 5.70E-07 | -0.1 | Whole Blood |
|  | rs6671847 | FCGR2A | RPS23P10 | 2.10E-05 | -0.1 | Whole Blood |
|  |  |  | RP11-122G18.11 | 2.20E-05 | 0.28 | Artery - Aorta |
|  | rs111487401 | TPD52 intron | none |  |  |  |
|  | rs1681087 | SARNP intron | none |  |  |  |
|  | rs12547167 | FAM167A | FAM167A | 2.50E-12 | 0.32 | Whole Blood |
|  |  |  | FAM167A | 3.60E-07 | 0.44 | Spleen |
|  | rs56317458 | CASP3 | CENPU | 3.50E-09 | -0.36 | Artery - Tibial |
|  |  |  | CENPU | 1.20E-08 | -0.36 | Heart - Left Ventricle |
|  |  |  | PRIMPOL | 4.80E-06 | 0.19 | Artery - Tibial |

UAP1L1: UDP-N-acetylglucosamine pyrophosphorylase 1 like 1 (Ubiquitous expression in spleen (RPKM 6.4), thyroid (RPKM 5.1) and 25 other tissues), SAPCD2: suppressor APC domain containing 2 (Broad expression in brain (RPKM 4.5), colon (RPKM 2.8) and 17 other tissues)

DPP7: dipeptidyl peptidase 7 (a post-proline cleaving aminopeptidase expressed in quiescent lymphocytes.), NPDC1: (neural proliferation, differentiation and control 1), MAN1B1-AS1: mannosidase alpha class 1B member 1 –antisense RNA1, ITPKC: inositol-trisphosphate 3-kinase C, EGLN2: egl-9 family hypoxia inducible factor 2, RPS23P10: ribosomal protein S23 pseudogene 10, RP11-122G18.11: AL831711.1 (Clone-based (Ensembl) gene), FAM167A: family with sequence similarity 167 member A, CASP3: caspase 3, CENPU: centromere protein U, PRIMPOL: primase and DNA directed polymerase.

**Supplementary Table 4.** FUMA / CADD / Regulome results

Functional Mapping and Annotation of SNVs in LD (r2>0.6) with associated SNVs in Table 2 and 3 with p<1.0E-05 (<https://fuma.ctglab.nl/>)

Excel file attached

**Supplementary Table 5.** The top 20 interactions of the CAA locus, ranked by their normalised contact frequency.


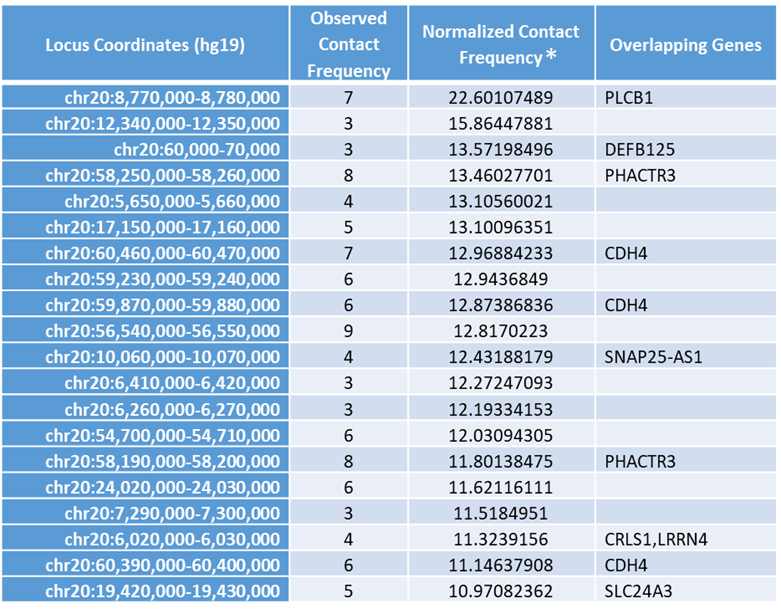


Regions were ranked using a “one-vs-all” strategy based on their normalized Hi-C contact frequency with our CAA locus, see Methods above for further details. *: Detailed explanation for normalised contact frequency is in supplemental methods.

As the CAA locus on 20q13.11 is intergenic and at least 50kb away from the nearest protein-coding gene, we hypothesised that it might act as distal regulatory element for genes affecting aneurysm development and severity. To test the hypothesis we analysed published data from a high-resolution 3D map of the human genome, generated using chromosome conformation capture techniques(ref. 12). We employed a “one-vs-all” strategy, in which we ranked every potential interacting region based on its normalized Hi-C contact frequency with our CAA locus.

The top interactor of the CAA locus is a 10kb region near the 3’ end of gene *PLCB1* (*chr20:8,770,000-8,780,000, build hg19*), encompassing exons 30 and 31. Inversely, when we examined all the interactors of this *PLCB1* subregion, the CAA locus was also the top hit (data not shown).

*PLCB1* encodes for the β1 isoform of Phospholipase C (PLC), a multifunctional enzyme implicated in cardiovascular function(ref. 13) and neuronal development(ref. 14). Furthermore, PLC-β1 is the major isoform expressed in heart tissue and has been shown to mediate cardiomyocyte hypertrophy(ref. 15). Genetic polymorphisms in *PLCB1* have been previously associated with CAA formation in Han Chinese Kawasaki patients(ref. 16). Follow-up functional experiments elucidated the role of *PLCB1* as a regulator of vascular inflammation.

**Supplementary Table 6**. Susceptibility meta-analysis results for SNVs with p<5x10-6 in the primary GWAS (score test followed by weighted z meta-analysis) which had p>5x10-6 with Wald test and inverse-variance meta-analysis. Qp is the p-value for a test for heterogeneity between the two studies. All effects are relative to the alternate allele, ref/alt alleles defined by the HRC reference panel.

| Chr. | Position (hg19) | SNV ID | Allele | | Cohort | | | | | | meta or (95% ci) | meta P | Qp |
| --- | --- | --- | --- | --- | --- | --- | --- | --- | --- | --- | --- | --- | --- |
|  |  |  |  |  | Khor 2012 | | | New | | |  |  |  |
|  |  |  | Reference | Alt. | OR | SE | P | OR | SE | P |  |  |  |
| 13 | 30184852 | rs117356586 | A | G | 1.242 | 0.149 | 1.5E-01 | 2.655 | 0.186 | 1.6E-07 | 1.67 (1.33,2.097) | 1.0E-05 | 1.4E-03 |
| 10 | 16998340 | rs4748329 | A | C | 1.669 | 0.175 | 3.3E-03 | 2.293 | 0.235 | 4.0E-04 | 1.87 (1.42,2.459) | 8.0E-06 | 2.8E-01 |
| 1 | 108004185 | rs148523506 | G | A | 1.644 | 0.170 | 3.4E-03 | 2.122 | 0.215 | 4.7E-04 | 1.81 (1.396,2.355) | 8.0E-06 | 3.5E-01 |

**Supplementary Table 7.** Top 50 genes from a MAGMA gene test for association with KD susceptibility. Columns specify the gene name, genomic position, the number of SNVs included in the gene test, effective number of SNVs, test statistic and p-value.

Excel file attached

**Supplementary Table 8**. Susceptibility meta-analysis results for SNVs identified from the EBI GWAS Catalog with p<5x10^-8^ for association with KD. Qp is the p-value for a test for heterogeneity between the European meta-analysis and the association reported on the EBI GWAS Catalog. Meta-analysis results are not reported for the Khor et al 2011 results as there are overlapping samples with our study. All effects are relative to the alternate allele, ref/alt alleles defined by the HRC reference panel.

|  |  |  |  | Allele | | European meta-analysis | | EBI GWAS Catalog | | meta or (95% ci) |  |  |  |
| --- | --- | --- | --- | --- | --- | --- | --- | --- | --- | --- | --- | --- | --- |
| Chr. | Position (hg19) | ID | Gene/Region | Reference | Alt. | OR | P | OR | P |  | meta.P | Qp | Reference |
| 8 | 11343680 | rs2254546 | *BLK, FAM167A* | A | G | 1.176167 | 0.071937 | 1.85 | 8.00E-21 | 1.176 (0.986,1.404) | 6.49E-18 | 0.000125 | (ref. 7) |
| 8 | 11343973 | rs2736340 | *BLK, FAM167A* | C | T | 1.276084 | 0.003252 | 1.538 | 9.00E-10 | 1.276 (1.085,1.501) | 4.85E-11 | 0.213948 | (ref. 17) |
| 6 | 32763514 | rs2857151 | *HLA-DQB2, HLA-DOB* | A | G | 0.939533 | 0.334723 | 1.47 | 5.00E-11 | 0.94 (0.828,1.066) | 2.45E-05 | 1.38E-06 | (ref. 7) |
| 1 | 1.61E+08 | rs1801274 | *FCGR2A* | A | G | 0.707206 | 1.08E-08 | 0.76 | 7.00E-11 | NA | NA | NA | (ref. 3) |
| 19 | 41281016 | rs2233152 | *MIA-RAB4B, MIA* | G | A | 1.476251 | 2.82E-07 | 1.52 | 4.00E-10 | NA | NA | NA | (ref. 3) |
| 20 | 44742064 | rs1569723 | *CD40, RPL13P2* | C | A | 1.109917 | 0.133373 | 1.415 | 6.00E-09 | 1.11 (0.969,1.272) | 7.03E-08 | 0.013024 | (ref. 17) |
| 20 | 44763284 | rs4813003 | *AL031687.1, CD40* | C | T | 0.959493 | 0.632341 | 0.71 | 5.00E-08 | 0.959 (0.81,1.137) | 2.78E-06 | 0.008553 | (ref. 7) |
| 4 | 1.86E+08 | rs2130392 | *CASP3, CENPU* | G | A | 1.183207 | 0.011074 | 1.42 | 3.00E-08 | 1.183 (1.039,1.347) | 8.34E-09 | 0.096601 | (ref. 7) |

**Supplementary Table 9.** Susceptibility meta-analysis results for SNVs identified from the EBI GWAS Catalog with 5x10^-8^<p<5x10^-6^ for association with KD. Qp is the p-value for a test for heterogeneity for difference in effect size between the European meta-analysis and the association reported on the EBI GWAS Catalog. All effects are relative to the alternate allele, ref/alt alleles defined by the HRC reference panel.

|  |  |  |  | Allele | | European meta-analysis | | EBI GWAS Catalog | | meta OR (95% ci) |  |  |  |
| --- | --- | --- | --- | --- | --- | --- | --- | --- | --- | --- | --- | --- | --- |
| Chr. | Position  (hg19) | ID | Gene/Region | Reference | Alt. | OR | P | OR | P |  | meta.P | Qp | Reference |
| 4 | 65170415 | rs7656244 | *TECRL* | C | A | 0.936338 | 0.396097 | 1.394 | 3.00E-07 | 0.936 (0.804,1.09) | 0.000711 | 0.000425 | (ref. 17) |
| 8 | 1.19E+08 | rs17667932 | *MED30* | T | C | 1.284088 | 0.024372 | 2.368 | 5.00E-07 | 1.284 (1.033,1.596) | 3.80E-06 | 0.010467 | (ref. 17) |
| 1 | 58757915 | rs527409 | *DAB1* | T | C | 1.123214 | 0.389612 | 0.344828 | 1.00E-06 | 1.123 (0.862,1.464) | 0.064588 | 2.34E-05 | (ref. 18) |
| 3 | 1.75E+08 | rs17531088 | *NAALADL2* | C | T | 1.125296 | 0.049062 | 1.43 | 1.00E-06 | 1.125 (1,1.266) | 3.76E-06 | 0.037904 | (ref. 19) |
| 2 | 64349202 | rs7604693 | *PELI1* | C | A | 1.017624 | 0.802523 | 0.37037 | 2.00E-06 | 1.018 (0.887,1.167) | 0.204095 | 2.51E-05 | (ref. 18) |
| 15 | 83726179 | rs1568657 | *AC024270.2, AC022558.2, AC024270.4, BTBD1* | A | G | 1.042458 | 0.598334 | 1.409 | 7.00E-06 | 1.042 (0.893,1.217) | 0.000322 | 0.022098 | (ref. 20) |
| 14 | 1.07E+08 | rs10129255 | *IGHV2-70, IGHV1-69D* | C | T | 1.099812 | 0.153467 | 1.315 | 7.00E-06 | 1.1 (0.965,1.253) | 1.87E-05 | 0.14097 | (ref. 20) |
| 3 | 1.61E+08 | rs9290065 | *PPM1L* | C | T | 0.948173 | 0.370392 | 0.471921 | 9.00E-06 | 0.948 (0.844,1.065) | 0.020527 | 0.000513 | (ref. 17) |

**Supplementary Table 10. Meta analysis of European GWASs and Japanese GWAS results for the CASP3 locus.** Results are for SNVs reported in Table 1 of Onouchi 2010 meta-analyzed with the Khor data and KD susceptibility data reported in this paper. (position hg19)


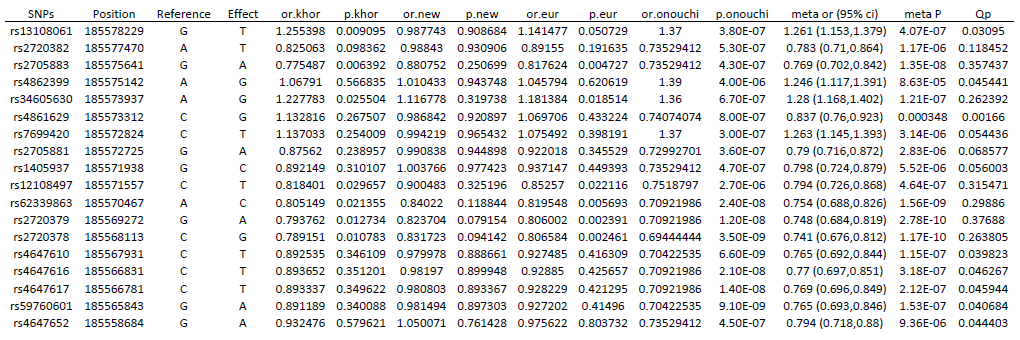


**Supplementary Table 11. Meta-analysis of European GWASs and Japanese GWAS results for the FAM167A/BLK locus**. Results are for SNVs reported in Supplementary Table 3 of Onouchi 2012 meta-analyzed with the Khor data and KD susceptibility data reported in this paper.


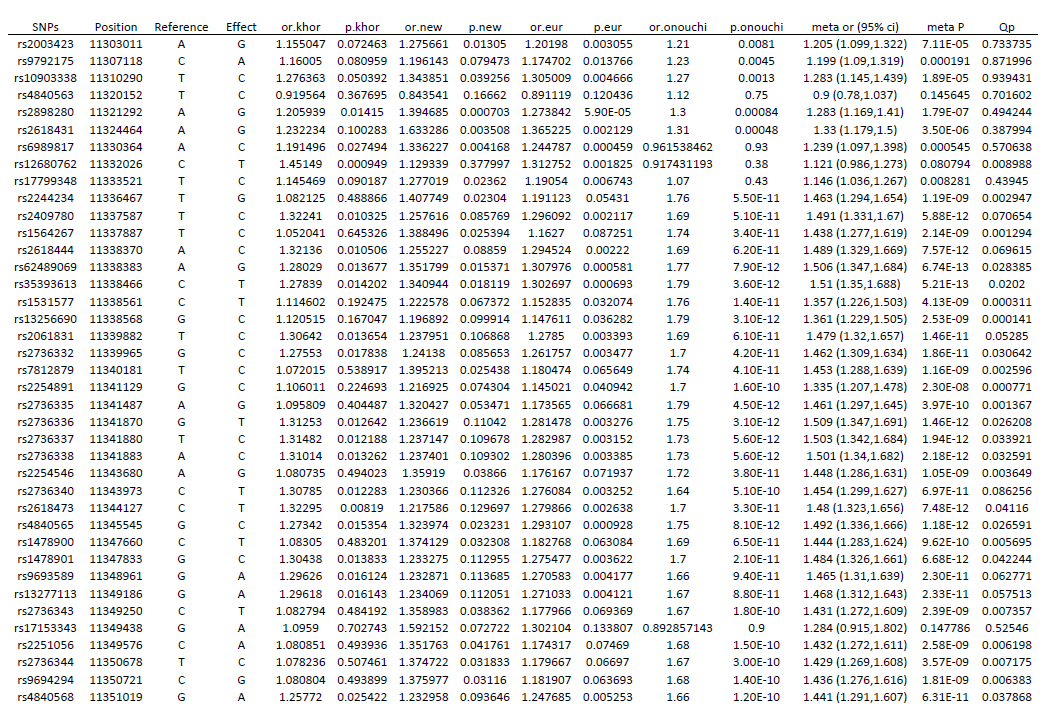


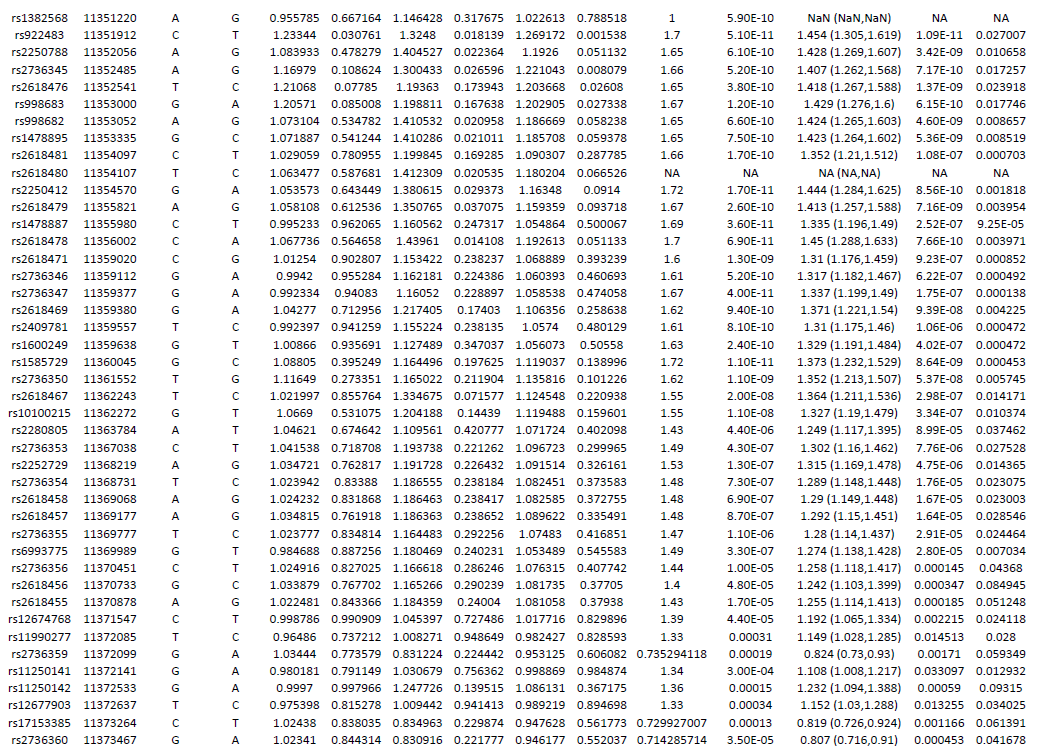


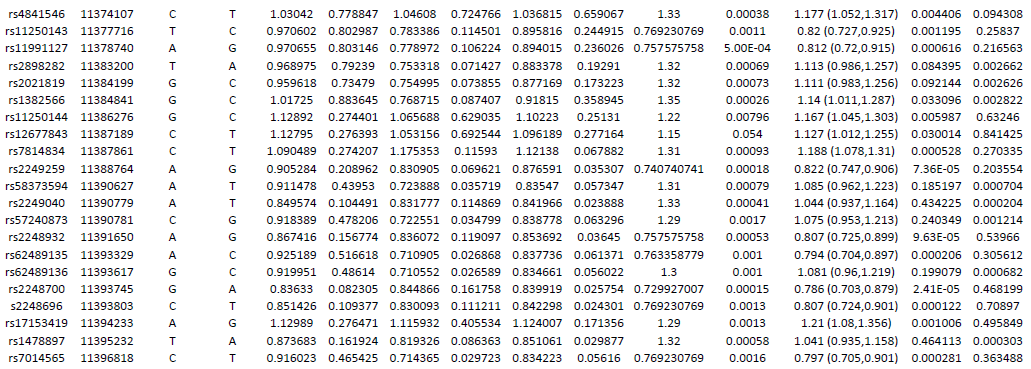


Position: hg19

**Supplementary Table 12.** Association of SNVs reported in Bugner et al for association with KD susceptibility in our European meta-analysis.

Excel file attached

**Supplementary Table 13**. Association of SNVs from the CAA GWAS with p<5x10^-6^ in the KD susceptibility GWAS and SNVs from the KD susceptibility GWAS with p<5x10^-6^ and the top SNVs in our analyses of the *CASP3* and FAM167A loci in the CAA GWAS. All effects are relative to the alternate allele, ref/alt alleles defined by the HRC reference panel.

|  |  |  |  |  | Allele | | Susceptibility Association | | CAA Association | |
| --- | --- | --- | --- | --- | --- | --- | --- | --- | --- | --- |
|  | Chr. | Position (hg19) | SNV ID | Gene region | Reference | Alt. | meta OR (95% ci) | meta P | or (95% ci) | p |
| SNVs associated with CAA | 20 | 41941664 | rs75202180 | Inter genic | A | G | 1.1 (0.9,1.3) | 0.59 | 4.9 (2.7-8.9) | 2.32E-08 |
|  | 5 | 169135662 | rs2449565 | *DOCK2* intron | A | G | 1.0 (0.9,1.1) | 0.60 | 2.3 (1.6-3.3) | 1.89E-06 |
|  | 10 | 73058469 | rs10762437 | *UNC5B* intron | A | G | 1.2 (1.0,1.4) | 0.03 | 2.5 (1.7-3.8) | 3.48E-06 |
|  | 3 | 106262305 | rs35932034 | Inter genic | T | C | 0.9 (0.7,1.1) | 0.27 | 0.2 (0.1-0.4) | 3.71E-06 |
|  | 8 | 129294118 | rs1989051 | Inter genic | T | C | 1.1 (0.9,1.2) | 0.42 | 2.2 (1.6-3.1) | 4.01E-06 |
| SNVs associated with susceptibility | 19 | 41248009 | rs3745213 | *ITPKC* | C | T | 1.6 (1.4-1.9) | 1.10E-09 | 0.8 (0.5,1.2) | 0.22 |
|  | 1 | 161478810 | rs6671847 | *FCGR2A* | G | A | 0.7 (0.6-0.8) | 2.50E-09 | 0.8 (0.6,1.2) | 0.30 |
|  | 12 | 56180764 | rs1681087 | *SARNP* | G | A | 1.7 (1.4-2.1) | 3.40E-06 | 0.5 (0.3,0.9) | 0.03 |
|  | 8 | 129294118 | rs2898280 | *FAM167A* | A | G | 1.3 (1.1,1.4) | 5.90E-05 | 0.9 (0.7,1.3) | 0.58 |
|  | 4 | 185541437 | rs56317458 | *CASP3* | C | T | 0.8 (0.7-0.9) | 2.00E-04 | 1.0 (0.7,1.5) | 0.94 |

**Supplementary Table 14.** Number of SNVs, cases and controls for each imputation run for the new susceptibility GWAS, the totals across all imputations runs of the new GWAS, the Khor GWAS and totals across both cohorts.

| Cohort | Genotyped SNVs | Cases | Controls |
| --- | --- | --- | --- |
| Imputation run 1 | 233819 | 216 | 1004 |
| Imputation run 2 | 468349 | 37 | 172 |
| Imputation run 3 | 227267 | 93 | 433 |
| Total New GWAS | - | 346 | 1609 |
| Khor | 488461 | 400 | 6101 |
| Total | - | 746 | 7700 |

**Supplementary Table 15**. GWAS Atlas PheWAS: SNV that had immunological traits with p<5.0E-05

Immunological or cardiovascular traits with p<5.0E-05 were searched for SNVs in Table 2 and Table 3. No cardiovascular trait had p<5.0E-05.

Excel file is attached.

**Supplementary Figure 1**. A Mapping of KD samples to 1000G.

Mapping of KD samples to 1000G. Selected and unselected KD subjects are denoted by (**+**) and (x), respectively. B. MDS 1 v MDS 2 for samples used in CAA GWAS. C, MDS 1 v MDS 2 for KD cases and vaccine controls used in the new susceptibility GWAS. D. SMDS 3 v MDS 4 for KD cases and vaccine controls used in the new susceptibility GWAS.

B.

A.


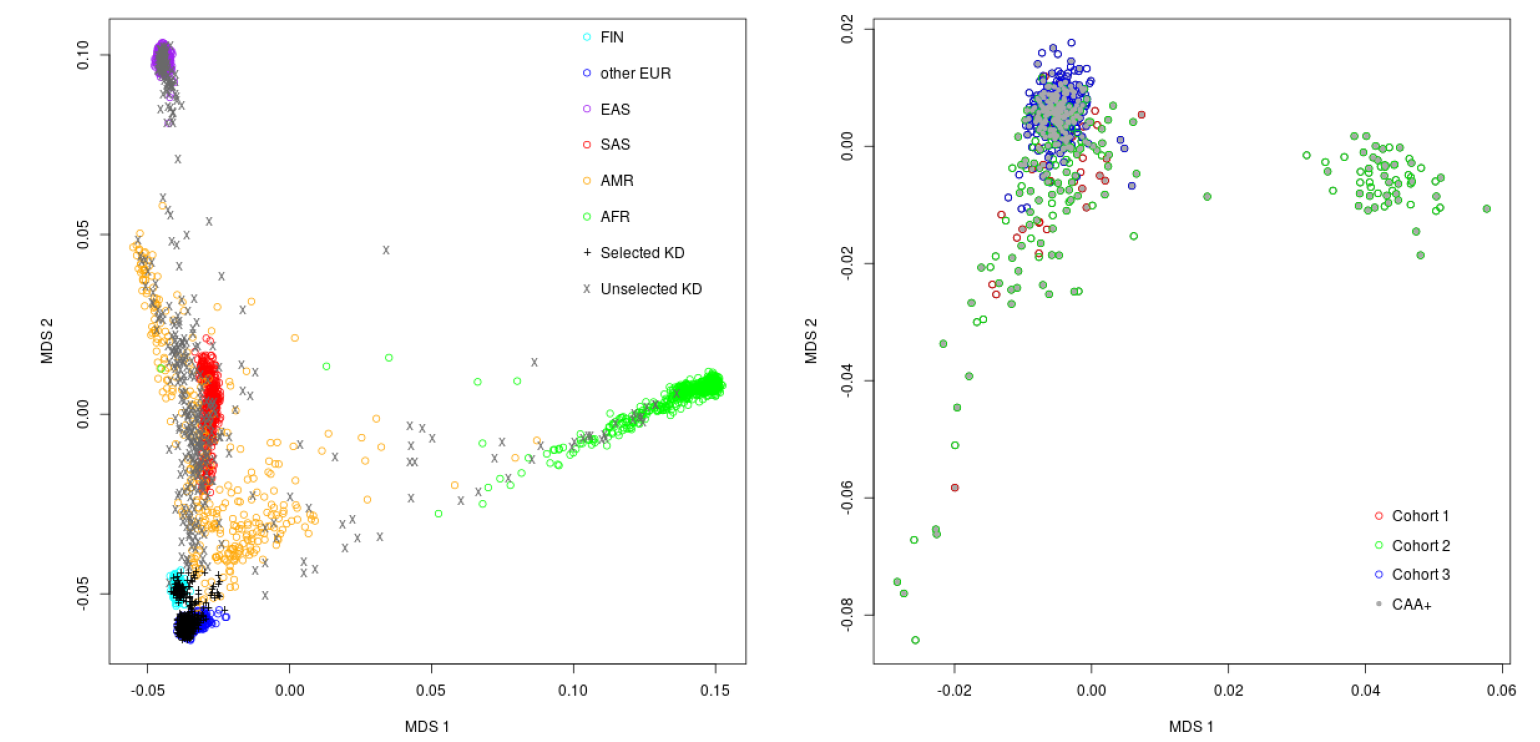

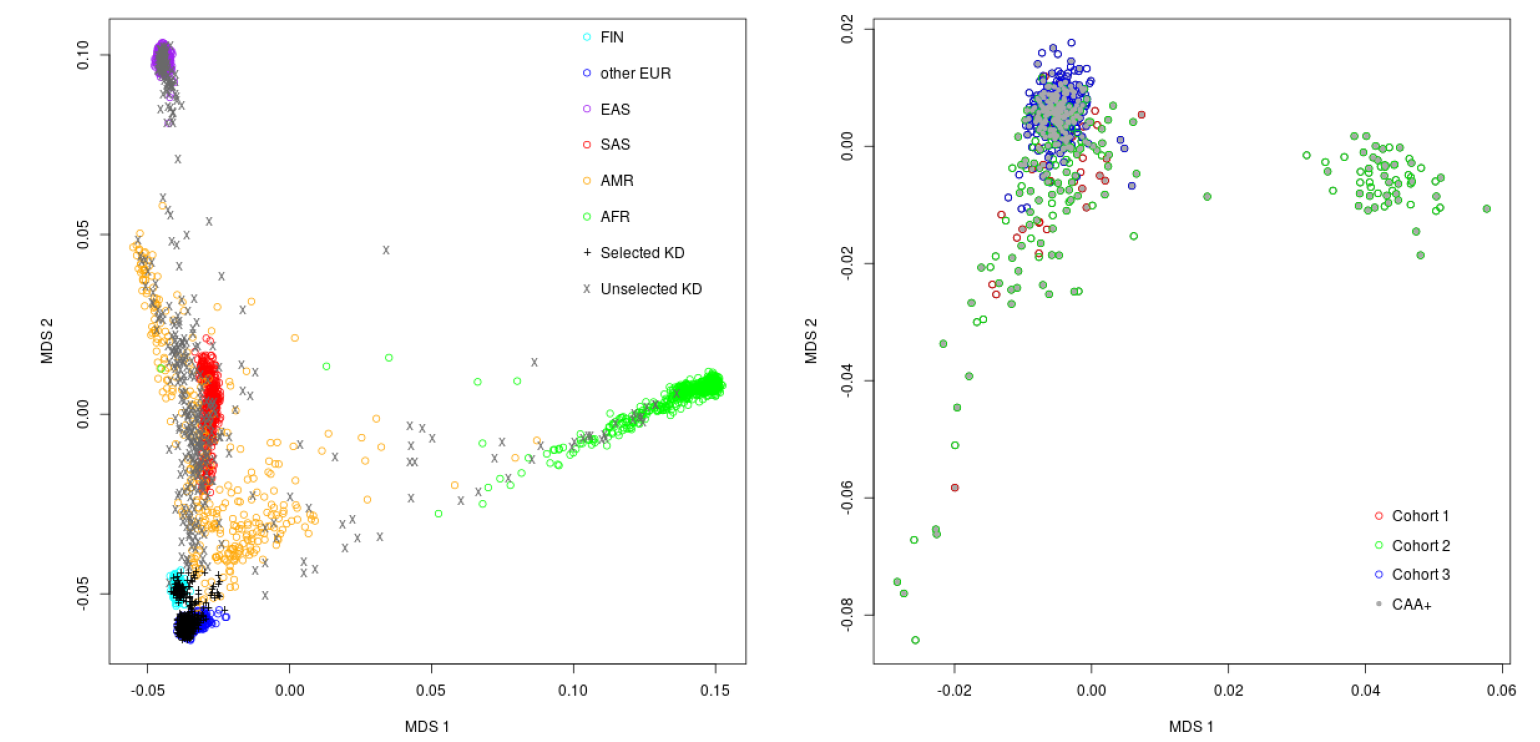


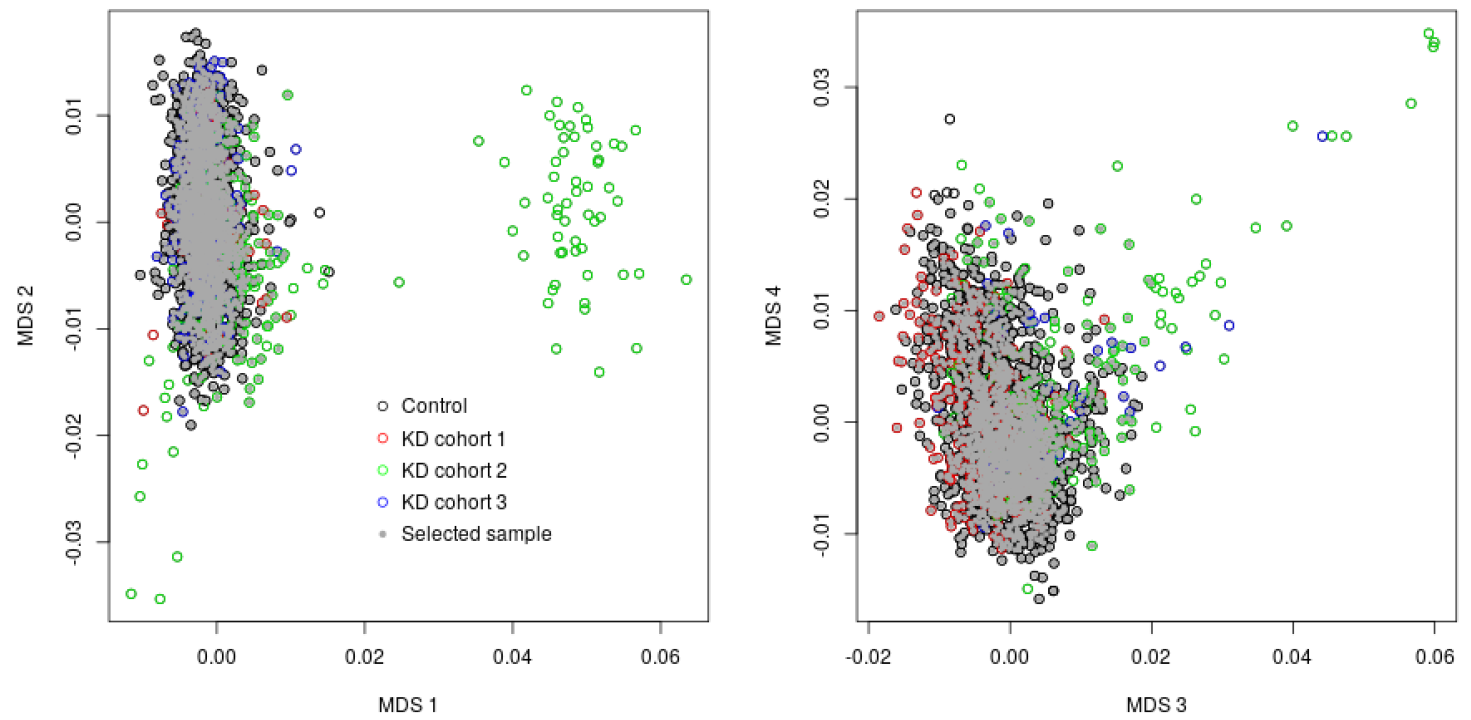


D.

C.

**Supplementary Figure 2.** QQ-plot of CAA GWAS

**Supplementary Figure 3.** Regional association plots for loci showing suggestive association with CAA risk with p <5x10^-6^

1. Chr. 9 MAN1B1 region. B. Chr. 5 DOCK2 region. C. Chr. 10 UNC3B region. D. Chr. 3 intergenic region. E. Chr. 8 intergenic region.

Position: hg19

1. B.


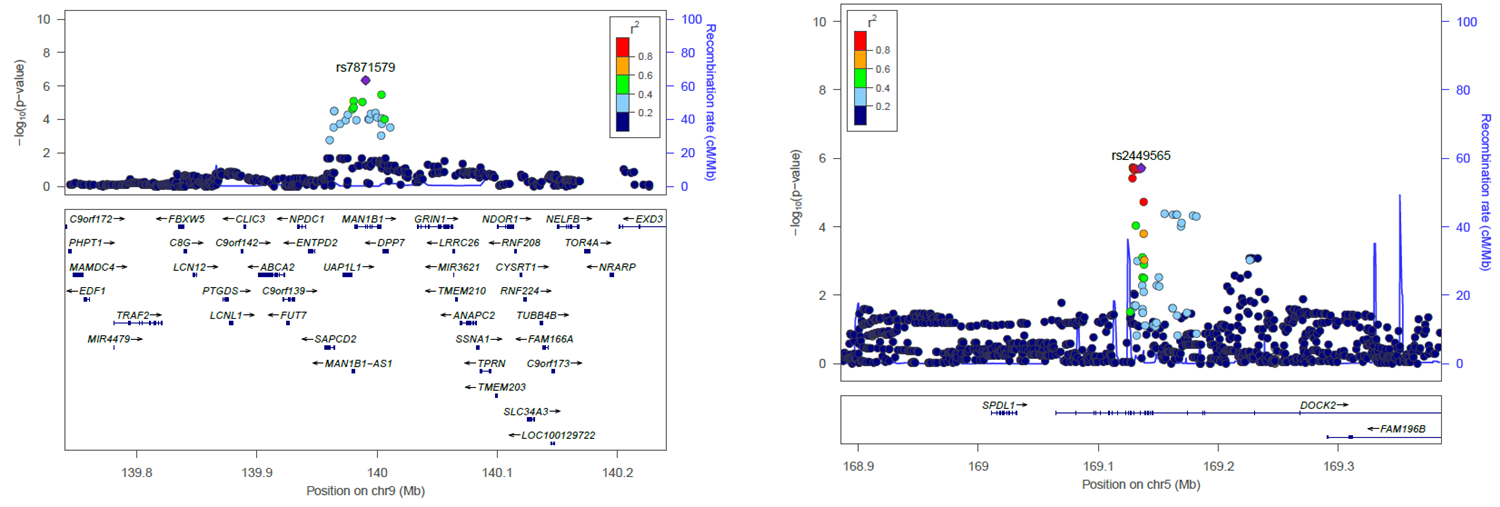


**Supplementary Figure 3.**

C. D.


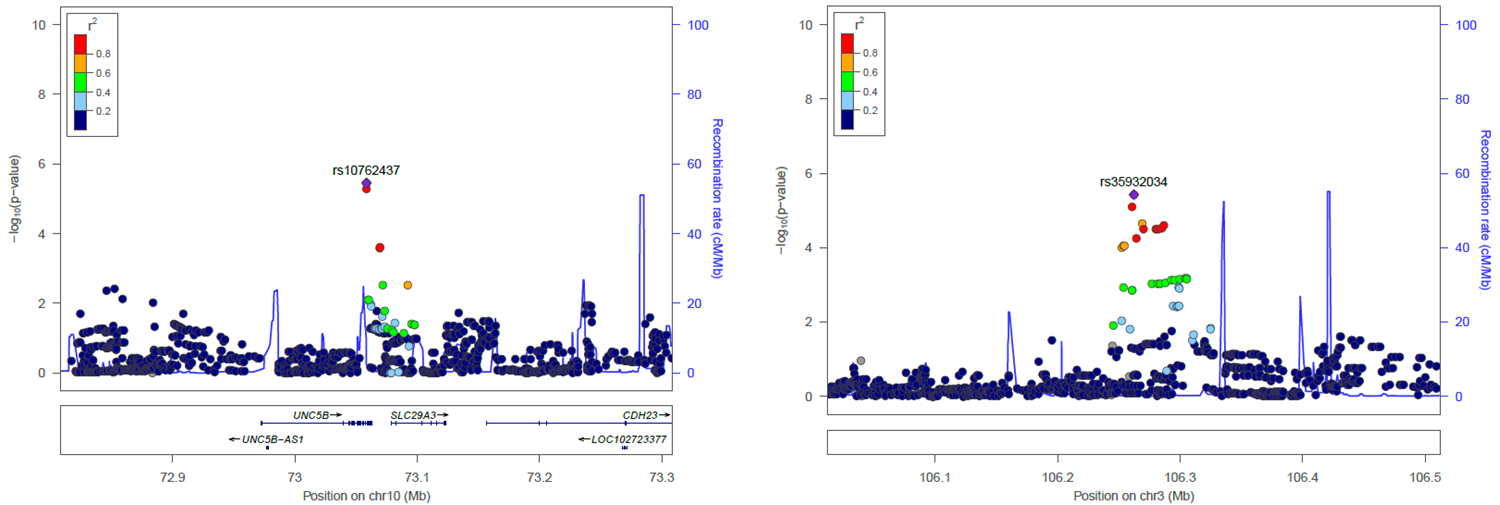


**Supplementary Figure 3.**

E.


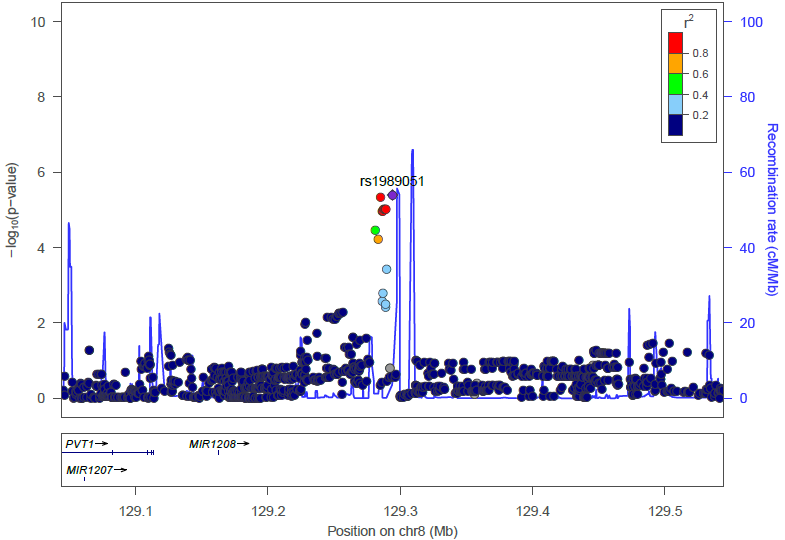


**Supplementary Figure 4**. QQ-plots for susceptibility GWASs and meta-analysis


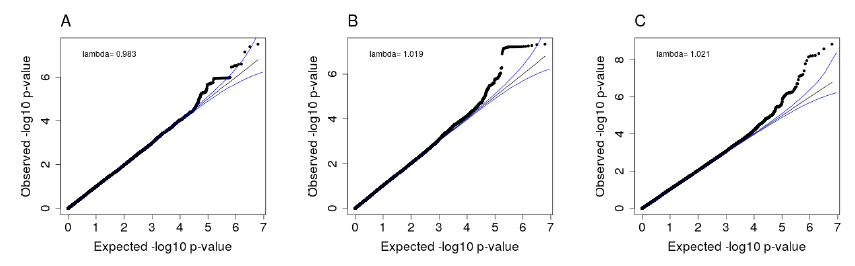


A: Khor GWAS.

B: GWAS using new samples and vaccine controls.

C: Meta-analysis

**Supplementary Figure 5**. Regional association plots for variants associated with KD susceptibility.

Results use score test results for the new susceptibility cohort and weighted Z meta-analysis. Position hg19

A. B.


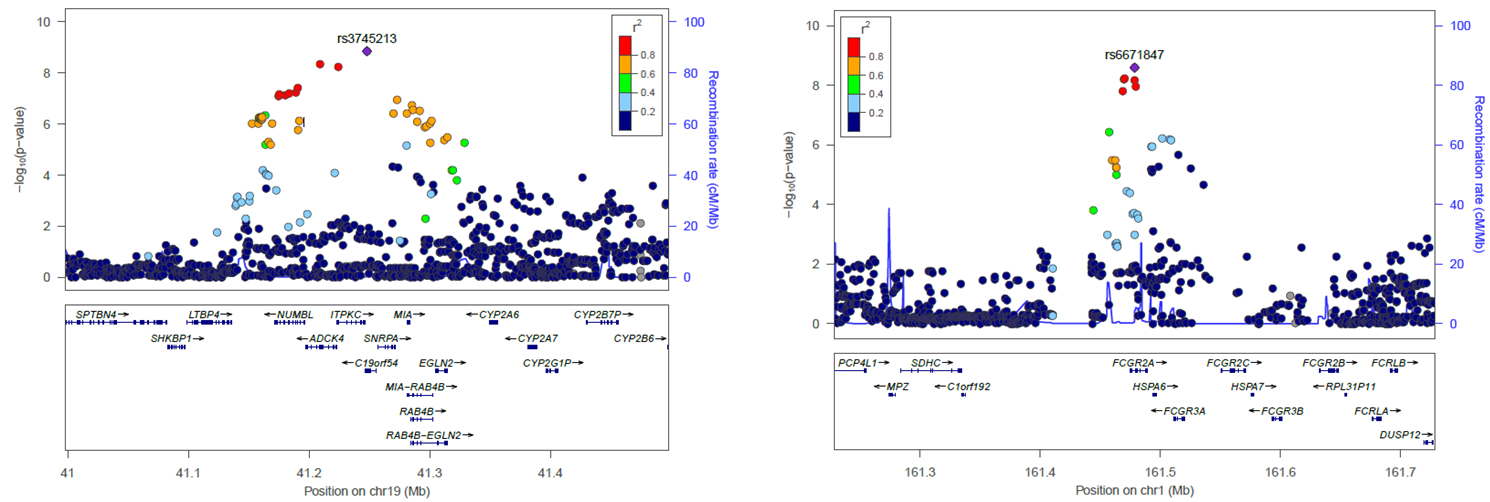


**Supplementary Figure 5.**

C. D.


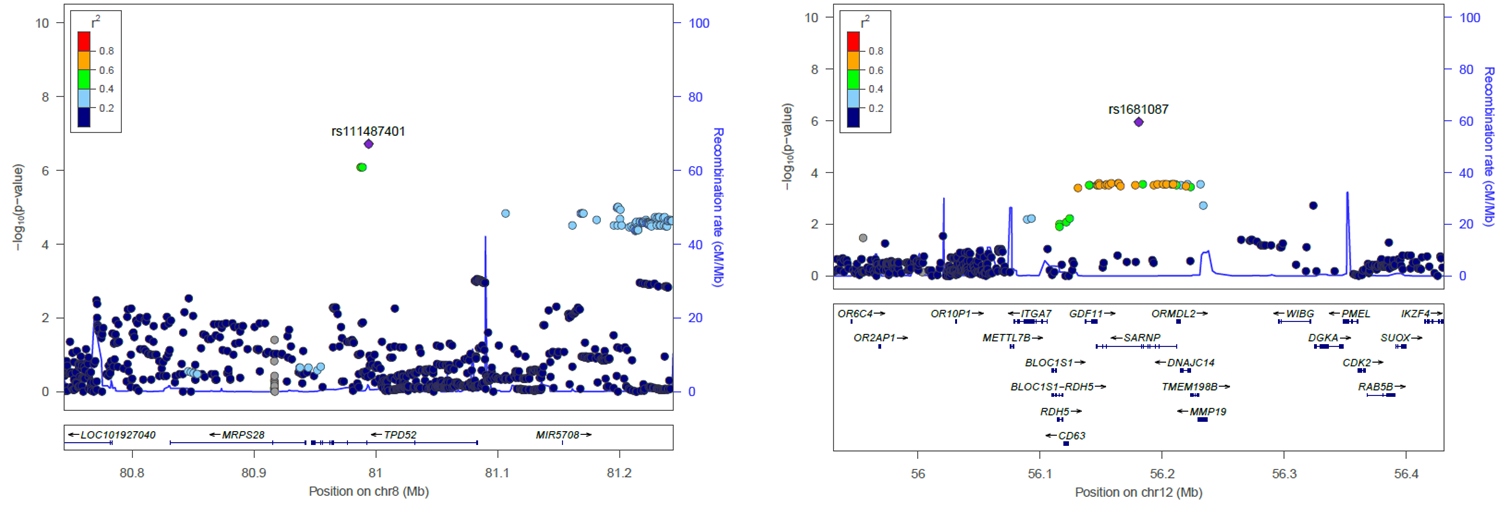


**Supplementary Figure 5.**

E. F.


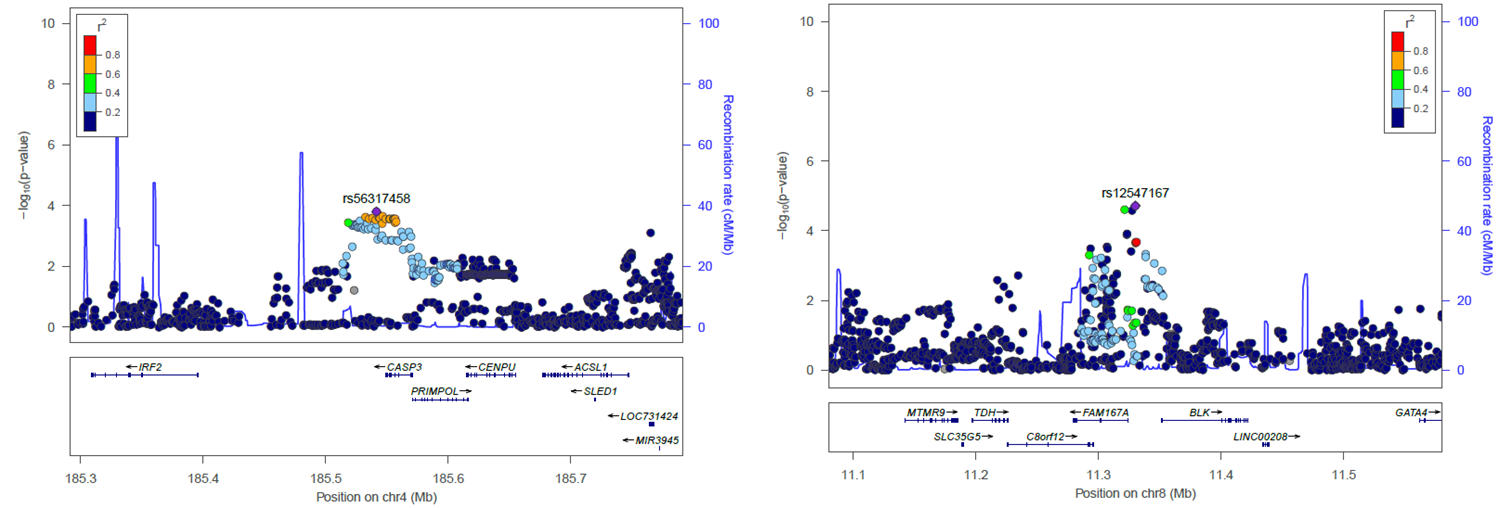


**Supplementary Figure 6.** Regional association plots for association with KD risk at the CASP3 locus. Position hg19

A: Data taken from Onouchi 2010(ref. 6) for a GWAS in a Japanese cohort.

B: Meta-analysis of Japanese cohort and European data used in this paper.


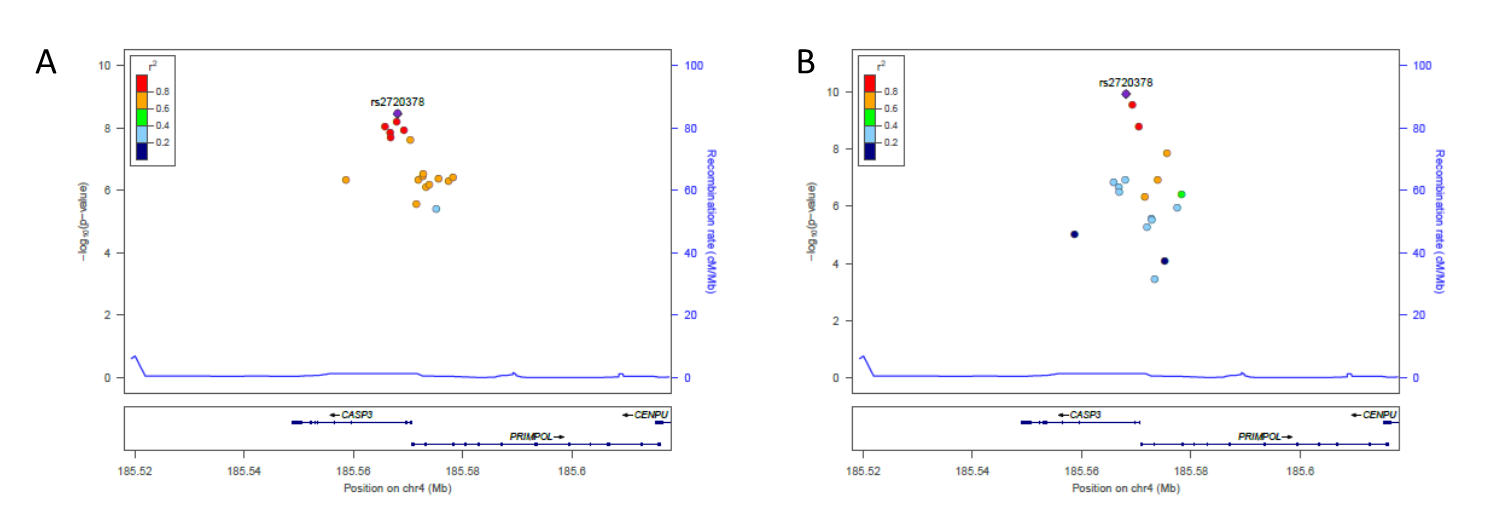


**Supplementary Figure 7**. Regional association plots for association with KD risk at the FAM167A/BLK locus. Position hg19

A: Data taken from Onouchi 2012 for a GWAS in a Japanese cohort.

B: Meta-analysis of Japanese cohort and European data used in this paper.


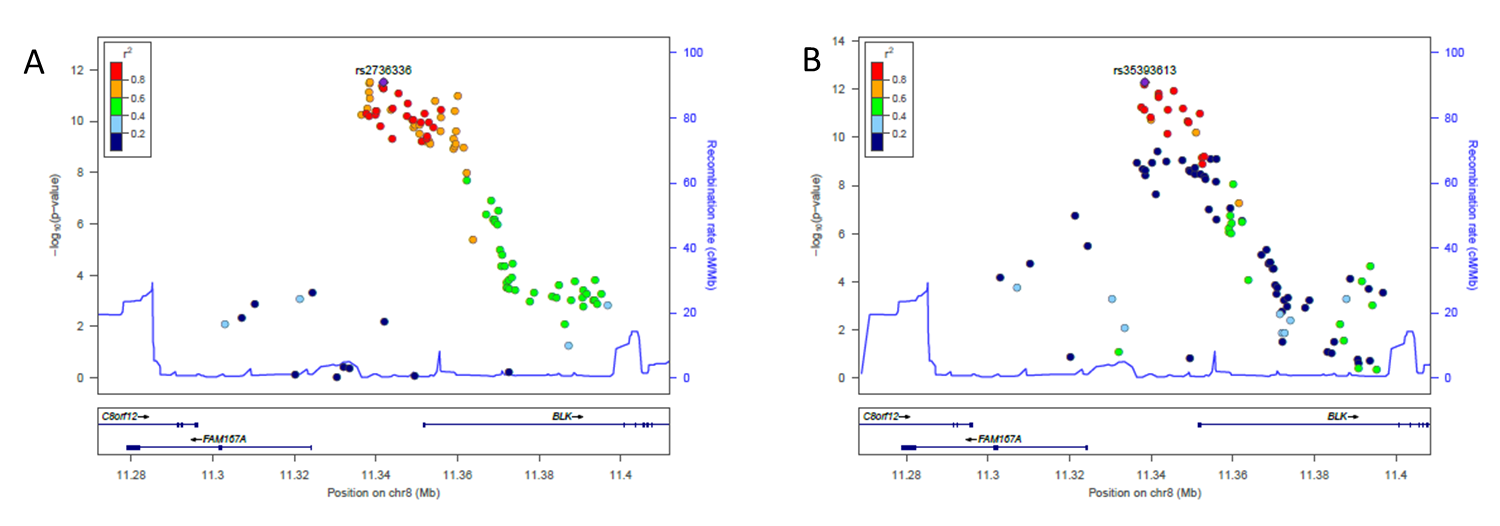


**Supplementary** References

1. Das S, Forer L, Schonherr S, Sidore C, Locke AE, Kwong A, et al. Next-generation genotype imputation service and methods. Nat Genet. 2016;48:1284-7.

2. O'Connor D, Png E, Khor CC, Snape MD, Hill AVS, van der Klis F, et al. Common Genetic Variations Associated with the Persistence of Immunity following Childhood Immunization. Cell Rep. 2019;27:3241-53 e4.

3. Khor CC, Davila S, Breunis WB, Lee YC, Shimizu C, Wright VJ, et al. Genome-wide association study identifies FCGR2A as a susceptibility locus for Kawasaki disease. Nat Genet. 2011;43:1241-6.

4. Yang J, Zaitlen NA, Goddard ME, Visscher PM, Price AL. Advantages and pitfalls in the application of mixed-model association methods. Nat Genet. 2014;46:100-6.

5. Ma C, Blackwell T, Boehnke M, Scott LJ, Go TDi. Recommended joint and meta-analysis strategies for case-control association testing of single low-count variants. Genet Epidemiol. 2013;37:539-50.

6. Onouchi Y, Ozaki K, Buns JC, Shimizu C, Hamada H, Honda T, et al. Common variants in CASP3 confer susceptibility to Kawasaki disease. Hum Mol Genet. 2010;19:2898-906.

7. Onouchi Y, Ozaki K, Burns JC, Shimizu C, Terai M, Hamada H, et al. A genome-wide association study identifies three new risk loci for Kawasaki disease. Nat Genet. 2012;44:517-21.

8. Bulik-Sullivan BK, Loh PR, Finucane HK, Ripke S, Yang J, Schizophrenia Working Group of the Psychiatric Genomics C, et al. LD Score regression distinguishes confounding from polygenicity in genome-wide association studies. Nat Genet. 2015;47:291-5.

9. Gazal S, Finucane HK, Furlotte NA, Loh PR, Palamara PF, Liu X, et al. Linkage disequilibrium-dependent architecture of human complex traits shows action of negative selection. Nat Genet. 2017;49:1421-7.

10. Skochko SM, Jain S, Sun X, Sivilay N, Kanegaye JT, Pancheri J, et al. Kawasaki Disease Outcomes and Response to Therapy in a Multiethnic Community: A 10-Year Experience. J Pediatr. 2018;203:408-15 e3.

11. Lee SH, Goddard ME, Wray NR, Visscher PM. A better coefficient of determination for genetic profile analysis. Genet Epidemiol. 2012;36:214-24.

12. Rao SS, Huntley MH, Durand NC, Stamenova EK, Bochkov ID, Robinson JT, et al. A 3D map of the human genome at kilobase resolution reveals principles of chromatin looping. Cell. 2014;159:1665-80.

13. Tappia PS, Singal T. Phospholipid-mediated signaling and heart disease. Subcell Biochem. 2008;49:299-324.

14. Spires TL, Molnar Z, Kind PC, Cordery PM, Upton AL, Blakemore C, et al. Activity-dependent regulation of synapse and dendritic spine morphology in developing barrel cortex requires phospholipase C-beta1 signalling. Cereb Cortex. 2005;15:385-93.

15. Filtz TM, Grubb DR, McLeod-Dryden TJ, Luo J, Woodcock EA. Gq-initiated cardiomyocyte hypertrophy is mediated by phospholipase Cbeta1b. FASEB J. 2009;23:3564-70.

16. Lin YJ, Chang JS, Liu X, Tsang H, Chien WK, Chen JH, et al. Genetic variants in PLCB4/PLCB1 as susceptibility loci for coronary artery aneurysm formation in Kawasaki disease in Han Chinese in Taiwan. Sci Rep. 2015;5:14762.

17. Lee YC, Kuo HC, Chang JS, Chang LY, Huang LM, Chen MR, et al. Two new susceptibility loci for Kawasaki disease identified through genome-wide association analysis. Nat Genet. 2012;44:522-5.

18. Kim JJ, Hong YM, Sohn S, Jang GY, Ha KS, Yun SW, et al. A genome-wide association analysis reveals 1p31 and 2p13.3 as susceptibility loci for Kawasaki disease. Hum Genet. 2011;129:487-95.

19. Burgner D, Davila S, Breunis WB, Ng SB, Li Y, Bonnard C, et al. A genome-wide association study identifies novel and functionally related susceptibility Loci for Kawasaki disease. PLoS Genet. 2009;5:e1000319.

20. Tsai FJ, Lee YC, Chang JS, Huang LM, Huang FY, Chiu NC, et al. Identification of novel susceptibility Loci for kawasaki disease in a Han chinese population by a genome-wide association study. PLoS One. 2011;6:e16853.
